# Therapeutic potential and molecular mechanisms of citrus flavonoids in hepatocellular carcinoma

**DOI:** 10.3389/fphar.2026.1848561

**Published:** 2026-08-25

**Authors:** Xiaoyan Zhu, Tao Zhang, Ting Gu, Hai Su, Yanyan Ren, Tong Cui

**Affiliations:** 1 Karamay Central Hospital, Karamay, Xinjiang, China; 2 The First Affiliated Hospital of Xinjiang Medical University, Urumqi, Xinjiang, China; 3 The First Jikun Hospital of Xinjiang Uygur Autonomous Region, Urumqi, Xinjiang, China

**Keywords:** hepatocellular carcinoma, hesperetin, hesperidin, metabolic reprogramming, naringenin, therapeutic potential

## Abstract

Citrus-derived flavonoids, particularly naringenin, hesperidin, and hesperetin, have emerged as promising bioactive compounds with potential therapeutic relevance in hepatocellular carcinoma (HCC). This review provides a focused synthesis of current preclinical evidence regarding their anticancer properties and underlying molecular mechanisms in experimental HCC models. These flavonoids exert broad anticancer effects, including inhibition of cell proliferation, induction of apoptosis, reduction of oxidative stress, and suppression of tumor progression and metastasis. These activities are mediated through modulation of key oncogenic signaling pathways, which are critically involved in hepatocarcinogenesis. In addition, citrus flavonoids influence mitochondrial function and cellular redox homeostasis, thereby contributing to programmed cell death and metabolic regulation. Preclinical studies further indicate that these compounds may enhance the efficacy of conventional chemotherapeutic agents, including cisplatin, doxorubicin, and sorafenib, while exhibiting reduced toxicity toward normal cells. Moreover, nanoformulation-based delivery systems have been shown to improve their bioavailability and therapeutic performance in experimental models. *In vivo* evidence supports their hepatoprotective effects, with reductions in tumor burden and attenuation of inflammation and fibrosis. Collectively, citrus flavonoids represent promising adjunctive candidates for HCC therapy; however, clinical validation remains limited, and further translational studies are required to confirm their therapeutic applicability in humans.

## 1 Introduction

Hepatocellular carcinoma (HCC) represents the predominant form of primary liver neoplasia, constituting approximately 75%–85% of all hepatic tumors and serving as a significant contributor to cancer-associated mortality on a global scale (Bhardwaj et al., 2026). The incidence of HCC continues to escalate, largely attributable to the rising prevalence of chronic hepatic conditions including viral hepatitis, alcohol-induced liver damage, and metabolic syndromes (Huang et al., 2023). Notwithstanding advancements in diagnostic and therapeutic modalities, HCC is frequently identified at more advanced stages, leading to unfavorable prognoses and constrained survival rates (Llo et al., 2003). Existing therapeutic strategies comprising surgical resection, liver transplantation, locoregional interventions, and systemic therapies such as targeted agents, remain inadequate due to elevated rates of recurrence, development of drug resistance, and adverse effects associated with treatment, thereby highlighting the pressing necessity for innovative and more efficacious therapeutic approaches (Sapisochin and Bruix, 2017; Mauro et al., 2023). A pivotal characteristic of HCC advancement is metabolic reprogramming, a phenomenon wherein neoplastic cells alter their metabolic pathways to facilitate accelerated proliferation, survival, and adaptation to a deleterious tumor microenvironment (Foglia et al., 2023).

This encompasses augmented aerobic glycolysis, modified lipid metabolism, dysregulated amino acid metabolism, and redox perturbation (Schirrmacher, 2020). Such metabolic modifications are meticulously regulated by interrelated signaling cascades, constituting a complex network that empowers tumor cells to satisfy heightened bioenergetic and biosynthetic requisites while fostering proliferation, metastasis, and immune evasion. Importantly, metabolic reprogramming not only propels tumor progression but also plays a crucial role in therapeutic resistance, rendering it a compelling target for innovative anticancer strategies (Liu et al., 2024; Chen et al., 2025). Natural bioactive compounds, particularly dietary flavonoids, have gained attention as potential anticancer agents due to their ability to modulate multiple signaling pathways with relatively low toxicity (Liskova et al., 2021; Naeem et al., 2022; Sharma et al., 2026). Among these compounds, flavonoids sourced from dietary origins have exhibited remarkable potential in the realms of cancer prevention and therapeutic intervention (Farhan et al., 2023; Montané et al., 2020). These bioactive entities display a range of biological effects, encompassing antioxidant, anti-inflammatory, and antiproliferative properties, and have been evidenced to disrupt critical signaling pathways that are instrumental in tumorigenesis and tumor progression. In addition, flavonoids may also influence metabolic reprogramming, suggesting their potential as complementary agents alongside conventional therapies (Wang et al., 2024a; Navarro et al., 2022).

Citrus flavonoids, specifically naringenin (NAR), hesperidin (HSD), and hesperetin (HST), have surfaced as formidable candidates in this domain owing to their significant anticancer attributes and their capacity to concurrently target multiple hallmarks of cancer (Pandey and Khan, 2021; Sharma et al., 2025). These phytochemicals have been documented to modulate pivotal signaling pathways such as AMP-activated protein kinase (AMPK), Wnt/beta-catenin (Wnt/β-catenin), mitogen-activated protein kinase (MAPK), and Janus kinase/signal transducer and activator of transcription (JAK/STAT), which are intimately associated with metabolic regulation, cellular survival, and tumor advancement (Wang et al., 2024b; Shine et al., 2018; Wu et al., 2025a; Lin et al., 2015). Furthermore, they possess the ability to affect essential biological processes, including apoptosis, ferroptosis, angiogenesis, and tumor immunity, thereby underscoring their potential as multifunctional therapeutic agents. Preclinical evidence also suggests potential synergy with conventional chemotherapeutic agents, along with acceptable safety profiles in experimental models. Among the numerous phytochemicals investigated in HCC, citrus-derived flavonoids such as NAR, HSD, and HST have gained increasing attention because of their multifaceted anticancer activities, favorable safety profiles, and capacity to modulate key oncogenic signaling pathways. Therefore, the present review specifically focuses on these structurally related citrus flavonoids and summarizes their therapeutic potential and underlying molecular mechanisms in HCC progression and treatment.

This comprehensive review aims to critically evaluate the therapeutic potential of citrus-derived flavonoids in HCC, with a particular focus on their roles in modulating key oncogenic signaling pathways and metabolic processes associated with tumor progression. Specifically, the review summarizes current preclinical evidence on the effects of NAR, HSD, and HST on cancer cell proliferation, apoptosis, oxidative stress, and metastasis in HCC models. In addition, special attention is given to their ability to regulate major signaling pathways involved in hepatocarcinogenesis, including phosphoinositide 3-kinase/protein kinase B (PI3K/Akt), Wnt/β-catenin, MAPK, and JAK2/STAT3. The review also highlights their potential to enhance the efficacy of conventional chemotherapeutic agents and their improved performance through nanoformulation strategies. Finally, this work discusses the current translational limitations and future perspectives necessary to bridge the gap between preclinical findings and clinical application in HCC therapy.

## 2 HCC: molecular pathogenesis and metabolic reprogramming

### 2.1 Epidemiology and risk factors

HCC constitutes approximately 75%–85% of primary hepatic malignancies and persists as a significant contributor to cancer-associated mortality on a global scale (Sung et al., 2021). The worldwide prevalence of HCC is on an upward trajectory, particularly in areas such as East Asia and sub-Saharan Africa; however, a rising incidence has also been documented in Western nations, attributable to metabolic disorders (Llovet et al., 2021). The principal risk factors associated with HCC encompass chronic infections caused by the hepatitis B virus and hepatitis C virus, which collectively account for the predominant proportion of cases worldwide (McGlynn et al., 2021). Significantly, the rising prevalence of obesity, insulin resistance, and type 2 diabetes has rendered non-alcoholic fatty liver disease (NAFLD) and its advanced form, non-alcoholic steatohepatitis, as prominent and emergent contributors to the etiology of HCC (Anstee et al., 2019).

### 2.2 Hallmarks of HCC

HCC manifests the quintessential characteristics of neoplasia as delineated by Hanahan and Weinberg, supplemented by liver-specific modifications (Hanahan, 2022). Prolonged proliferative signaling is often instigated by the activation of pathways including Wnt/β-catenin, MAPK, and PI3K/Akt (Llovet et al., 2021). The resistance to cellular demise, particularly through apoptosis and ferroptosis, constitutes a hallmark of HCC and significantly contributes to therapeutic resistance (Dixon et al., 2012; Tang et al., 2021). Furthermore, a pivotal hallmark of HCC encompasses the promotion of angiogenesis, predominantly mediated by vascular endothelial growth factor (VEGF), which facilitates tumor expansion within the hypoxic hepatic microenvironment (Forner et al., 2018). Moreover, immune evasion is instrumental, with neoplastic cells manipulating immune checkpoints and altering the tumor microenvironment to inhibit anti-tumor immune responses (Ringelhan et al., 2018). Metabolic reprogramming has emerged as a fundamental hallmark of HCC, permitting neoplastic cells to acclimatize to nutrient scarcity and maintain accelerated proliferation (Pavlova and Thompson, 2016).

### 2.3 Metabolic reprogramming in HCC

Metabolic reprogramming in HCC involves coordinated alterations in glucose, lipid, and mitochondrial metabolism, along with redox homeostasis. One of the most significant metabolic characteristics of HCC is the upregulation of aerobic glycolysis, commonly referred to as the Warburg effect, wherein neoplastic cells preferentially metabolize glucose to lactate, even in the presence of adequate oxygen levels (Vander Heiden et al., 2009). This metabolic alteration facilitates rapid production of adenosine triphosphate (ATP) and supplies precursors for various biosynthetic pathways. Key enzymes involved in glycolysis, including hexokinase 2 (HK2), pyruvate kinase M2, and lactate dehydrogenase A (LDHA), are frequently found to be upregulated in cases of HCC (Park and Hall, 2025). Altered lipid metabolism constitutes a significant characteristic of HCC, distinguished by an elevation in *de novo* lipogenesis coupled with a disruption in fatty acid oxidation. Enzymatic proteins such as fatty acid synthase (FASN) and acetyl-CoA carboxylase exhibit increased expression levels, thereby promoting membrane biosynthesis and energy storage (Jones and Infante, 2015; Flavin et al., 2010). Sterol regulatory element-binding proteins are integral to the modulation of lipogenic gene expression within the context of HCC (Cheng et al., 2022). Furthermore, dysregulated β-oxidation and lipid accumulation are implicated in tumor progression, particularly in cases of NAFLD-associated HCC (Anstee et al., 2019). HCC cells manifest heightened concentrations of reactive oxygen species (ROS), which can facilitate tumorigenesis by instigating DNA damage and activating oncogenic signaling cascades (Reczek and Chandel, 2015). Nonetheless, an overabundance of ROS may prove deleterious; consequently, neoplastic cells uphold a finely-tuned redox equilibrium. Antioxidant mechanisms, comprising glutathione (GSH), superoxide dismutase (SOD), and nuclear factor erythroid 2–related factor 2 (Nrf2), are frequently upregulated in HCC to mitigate oxidative stress (Dodson et al., 2019). Significantly, perturbation of redox homeostasis can elicit ferroptosis, a modality of iron-dependent cellular demise that is increasingly acknowledged as a viable therapeutic target in HCC (Tang et al., 2021).

## 3 Citrus flavonoids: chemistry, bioavailability, and pharmacokinetics

### 3.1 Structural characteristics

Citrus flavonoids are polyphenolic compounds primarily belonging to the flavanone subclass, characterized by a conserved C6–C3–C6 backbone consisting of two aromatic rings (A and B) linked through a heterocyclic pyran ring (C) (Panche et al., 2016). Structural variations among NAR, HST, and HSD critically influence their physicochemical properties, cellular uptake, molecular interactions, and biological activities in HCC. NAR, the aglycone form, contains hydroxyl groups at the 4′, 5, and seven positions, which contribute to its antioxidant capacity, redox regulation, and ability to form hydrogen-bond interactions with signaling proteins and kinases (Rani et al., 2016). These properties may facilitate modulation of oxidative stress-sensitive pathways involved in apoptosis, ferroptosis, and metabolic reprogramming. HST possesses a similar flavanone scaffold but contains a methoxy group at the 4′position, increasing lipophilicity and potentially enhancing membrane permeability and intracellular accumulation, thereby influencing interactions with mitochondrial and cytoplasmic signaling pathways. In contrast, HSD represents the glycosylated form of HST conjugated with the disaccharide rutinose, which improves aqueous solubility but may reduce passive membrane diffusion and alter bioavailability (Manach et al., 2004). Importantly, hydroxylation patterns, methoxylation, and glycosylation status can influence the affinity of these flavonoids toward molecular targets including PI3K/Akt, MAPK, nuclear factor kappa B (NF-κB), and Wnt/β-catenin signaling mediators. Through modulation of redox homeostasis, mitochondrial function, and kinase-dependent signaling, these structural features contribute to cellular and metabolic reprogramming in HCC cells, ultimately affecting proliferation, apoptosis, ferroptosis, inflammation, and tumor progression.

### 3.2 Absorption, metabolism, and bioavailability

The bioavailability of citrus flavonoids is typically constrained and is significantly influenced by their chemical structure as well as the processes of intestinal metabolism. Glycosylated flavonoids, such as HSD, necessitate hydrolysis facilitated by intestinal microbiota or enzymes like β-glucosidases to liberate the aglycone HST prior to its absorption (Manach et al., 2004). Both NAR and HST are taken up in the small intestine through mechanisms of passive diffusion and potentially via carrier-mediated transport (Rani et al., 2016). Subsequent to absorption, these compounds are subjected to extensive phase II metabolic processes within enterocytes and hepatocytes, which include glucuronidation, sulfation, and methylation, ultimately affecting their systemic circulation and biological activity (D'Archivio et al., 2010). The plasma levels of free aglycones are generally low, with their metabolites constituting the predominant circulating forms. Furthermore, first-pass metabolism and rapid elimination serve to restrict their bioavailability. Nevertheless, recent advancements in nanoformulations and delivery systems have demonstrated potential in improving the stability, absorption, and therapeutic efficacy of citrus flavonoids in liver cancer models.

### 3.3 Safety and toxicological profile

Citrus flavonoids, including NAR, HSD, and HST, are typically classified as safe substances, exhibiting low toxicity in both preclinical and clinical investigations (Rani et al., 2016). These phytochemicals demonstrate considerable tolerability owing to their natural presence in widely consumed fruits and their relatively minimal tendency for bioaccumulation. Both *in vitro* and *in vivo* research suggest that these compounds do not elicit notable cytotoxic effects in normal cells at concentrations that are pharmacologically relevant (Panche et al., 2016). Nonetheless, at elevated dosages, certain flavonoids may disrupt the activity of drug-metabolizing enzymes, particularly cytochrome P450 (CYP450) isoforms, which could potentially result in herb–drug interactions (D'Archivio et al., 2010). Furthermore, excessive consumption may modify hormone metabolism or present pro-oxidant effects under specific conditions. Toxicological assessments have indicated a broad therapeutic window; however, the availability of long-term safety data pertaining to human subjects remains inadequate. Consequently, additional investigations are warranted to determine optimal dosing regimens and evaluate potential risks associated with clinical applications in the treatment of HCC.

## 4 Molecular targets and signaling pathways modulated by NAR in HCC

### 4.1 *In vitro* evidence

**FIGURE 1:**
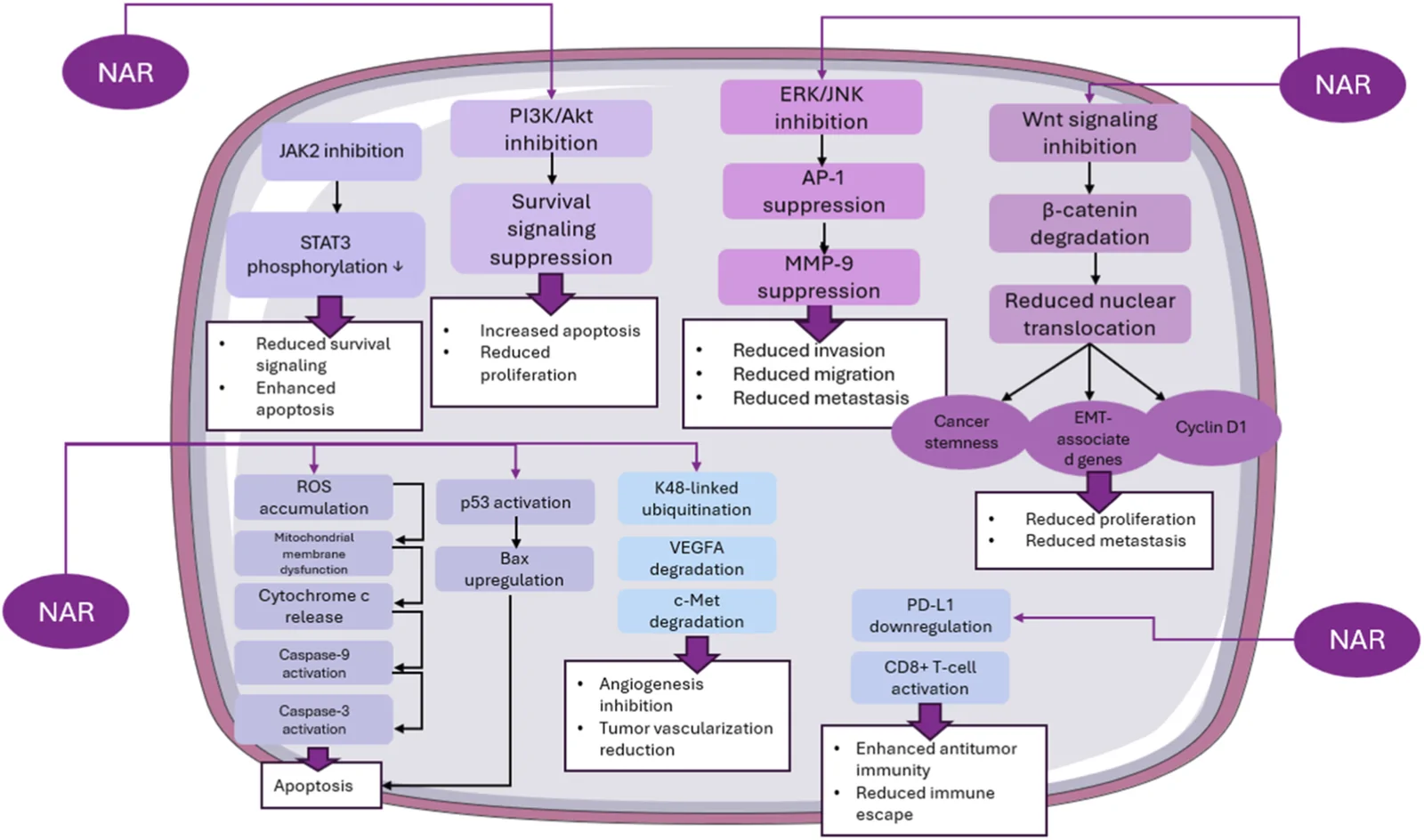
Molecular mechanisms of naringenin (NAR) in hepatocellular carcinoma (HCC). Schematic overview of the multitarget anticancer effects of NAR in HCC. NAR suppresses tumor progression by inhibiting major oncogenic signaling pathways, including Wnt/β-catenin, MAPK, PI3K/Akt, and JAK2/STAT3, leading to reduced proliferation and survival. It induces apoptosis via mitochondrial dysfunction, ROS accumulation, and activation of caspase-9/3, and also triggers autophagy-associated cell death. NAR inhibits metastasis and angiogenesis through NF-κB/AP-1 suppression, MMP-9 downregulation, and degradation of VEGFA and c-Met, while also enhancing antitumor immunity via PD-L1 suppression and CD8^+^ T-cell activation. Additionally, NAR modulates metabolic reprogramming through AMPK activation and promotes ferroptosis sensitivity. It restores redox balance by increasing antioxidant defenses (SOD, CAT, GPx, GSH). Nanoformulations improve NAR bioavailability, stability, and tumor targeting, enhancing its overall anticancer efficacy.

An *in vitro* study demonstrated that NAR inhibits proliferation, migration, and invasion of HCC cell lines while inducing apoptosis. The dominant mechanisms involve modulation of Wnt and MAPK signaling pathways through regulation of key molecular targets (IGFBP3, PGF, CA9, AKR1C3, KLK1, CHRNA7). Secondary mechanisms include alterations in prognostic markers such as CA9 and AKR1C3. These molecular events result in suppression of tumor-cell growth and enhanced apoptotic activity *in vitro* (Zhang et al., 2025) (Table 1; Figure 1). *In vitro* results in HepG2 cells showed that NAR nanoparticles significantly enhance cytotoxic efficacy compared with free NAR. The dominant mechanism is activation of p53-mediated apoptosis and autophagy (ATG5, LC3). Secondary mechanisms include improved cellular uptake, stability, and bioavailability. Importantly, nanoparticle formulation improves pharmacokinetic behavior by overcoming poor solubility and increasing intracellular drug accumulation, while reducing toxicity in normal cells, indicating superior translational potential compared with free NAR (Elwan et al., 2025). *In vitro* co-encapsulation of NAR with ibuprofen significantly enhanced anticancer activity. The dominant mechanism is synergistic induction of apoptosis and suppression of proliferation. Secondary mechanisms include inhibition of migration and angiogenesis. Nanoencapsulation improves drug delivery efficiency and bioavailability, resulting in lower IC50 values and enhanced cytotoxic effects compared with free compounds (Almoallim et al., 2024).

**TABLE 1:** Effects and molecular mechanisms of naringenin in hepatocellular carcinoma.

Model/System	Main effects	Molecular targets/Pathways	Key findings	Therapeutic implications	Study
*In vitro:* HCC cells, HUVECs *In vivo:* Mouse model, CAM assay	- Inhibits tumor growth - Suppresses angiogenesis - Enhances anti-tumor immunity	- VEGFA (degradation via K48 ubiquitination) - c-Met (degradation) - PD-L1 ↓ - CD8^+^ T cells↑	- Reduces endothelial tube formation and migration - Decreases neovascularization - Increases CD8^+^ T Cell infiltration and cytokines - Enhances ubiquitin-mediated degradation of VEGFA and c-Me	- Potential dual anti-angiogenic and immunotherapeutic agent - Synergistic effects with PD-L1 antibodies and bevacizumab	Wu et al. (2025b)
*In vitro:* MHCC-97H, Huh7 cells *In vivo:* Xenograft mouse model	- Inhibits proliferation, migration, invasion - Induces apoptosis	- Wnt signaling pathway - MAPK signaling pathway - Target genes: IGFBP3, PGF, CA9, AKR1C3, KLK1, CHRNA7	- Significant differential gene expression - CA9 and AKR1C3 identified as prognostic markers - Reduced tumor volume and weight *in vivo*	- Multi-target therapeutic potential - May serve as a regulator of oncogenic signaling pathways in HCC	Zhang et al. (2025)
*In vitro:* HepG2, WI38 cells	- Strong antiproliferative effect - Induces apoptosis and autophagy - Cell cycle arrest	- p53 ↑ - ATG5 ↑ - LC3 ↑	- Higher potency than free naringenin (lower IC50) - Induces late apoptosis (56.1%) - Arrests cells in G0/G1 and G2/M phases - Selective toxicity (no effect on normal cells)	- Improved bioavailability via nanoparticles - Enhances doxorubicin efficacy in cancer cells while reducing toxicity in normal cells - Promising nano-based therapeutic strategy	Elwan et al. (2025)
*In vitro:* HepG2 cells	- Strong antiproliferative effect - Anti-migratory and anti-angiogenic effects - Induces apoptosis	- Not specifically defined - Likely multi-target effects (inflammation and proliferation pathways)	- Lower IC50 for nano-encapsulated drugs vs. free forms - High encapsulation efficiency (NAR: ∼86%) - Synergistic interaction (CI < 1) - Enhanced inhibition of migration and angiogenesis	- Nano-co-delivery enhances bioavailability and efficacy - Synergistic combination therapy (IBU + NAR) - Promising multifunctional nanotherapeutic approach	Almoallim et al. (2024)
*In vitro:* Liver cancer cell lines *In vivo:* Xenograft mouse model	- Enhances ferroptosis - Increases lipid peroxidation - Inhibits tumor growth	- AMPK activation ↑ - PGC1α activation ↑ - Aerobic glycolysis ↓	- Synergistic effects with ferroptosis inducers (erastin, RSL3, sorafenib) - Increased sensitivity to ferroptosis- Effect reversed by AMPK/PGC1α inhibitors	- Sensitizer for ferroptosis-based therapy - Targets metabolic reprogramming in HCC - Potential combination with standard therapies (e.g., sorafenib)	Li et al. (2024)
*In vitro:* HepG2 cells	- Inhibits cell proliferation - Enhances cytotoxicity	- Not specifically defined - Likely apoptosis-related pathways	- Nanoparticles show significantly lower IC50 than free compounds - Naringenin NPs more effective than free form - Comparable enhancement observed with boswellic acids and curcumin	- Nanoparticle formulation improves anticancer efficacy - Supports use of natural compounds in nanomedicine - Potential combinational phytochemical therapy	Elnawasany et al. (2023)
*In vitro:* HepG2 cells	- Induces apoptosis - Increases oxidative stress (ROS) - Mitochondrial dysfunction	- ROS ↑ - JAK2/STAT3 inhibition ↓ - Caspase-3, Caspase-9 activation ↑	- Increased sub-G1 population and DNA fragmentation - Loss of mitochondrial membrane potential (ΔΨm) - Activation of intrinsic apoptotic pathway	- ROS-mediated apoptosis as a key mechanism - Targeting JAK2/STAT3 signaling in HCC - Potential oxidative stress-based therapeutic strategy	Zhang et al. (2023)
*In vitro:* HepG2 cells	- High dose: antiproliferative, pro-apoptotic - Low dose: cytoprotective	- ROS-mediated p53 pathway - Mitochondrial membrane potential (ΔΨm)	- High concentrations enhance tamoxifen efficacy (↑ apoptosis, ↓ migration/invasion) - Low concentrations antagonize tamoxifen effects - Biphasic (dual) response depending on dose	- Critical importance of dose optimization - Potential adjuvant with tamoxifen (at appropriate doses) - Highlights risk of unintended protective effects	Xu et al. (2022)
*In vitro and* *in vivo:* HCC cells, xenograft models	- Inhibits proliferation, migration, invasion - Suppresses cancer stemness - Reduces metastasis	- Wnt/β-catenin pathway ↓ - β-catenin degradation ↑ - GSK3β activation ↑ - HIF-1 activity ↓	- Reduced sphere formation and EMT - Downregulation of stemness markers (e.g., NANOG) - Increased sensitivity to anticancer drugs - Decreased tumor growth and lung metastasis	- Targets cancer stem cells in HCC - Enhances chemosensitivity - Promising derivative for anti-metastatic therapy	Kang et al. (2019)
*In vivo:* DEN/2AAF-induced HCC in Wistar rats	- Prevents hepatocarcinogenesis - Anti-inflammatory and antioxidant effects - Promotes apoptosis	- Oxidative stress markers (NO ↓, lipid peroxidation ↓) - Antioxidant enzymes (SOD, GPx, CAT ↑, GSH ↑) - IL-4, p53, Bcl-2 regulation	- Reduced liver tumor markers (AFP, CEA, CA19.9) - Improved liver function (ALT, AST, ALP, γ-GT) - Suppressed histopathological damage and inflammation	- Strong chemopreventive potential - Synergistic phytochemical therapy (naringenin + quercetin) - Targets oxidative stress and inflammation in HCC	Ahmed et al. (2022)
*In vitro:* HepG2, Huh-7, HA22T cells	- Suppresses invasion and metastasis - Reduces MMP-9 expression	- NF-κB ↓ - AP-1 ↓- ERK/JNK pathways ↓- PI3K/Akt pathway ↓	- Inhibition of MMP-9 transcription and secretion - Blockade of NF-κB nuclear translocation via IκB - Suppression of TPA-induced signaling cascades	- Anti-metastatic potential - Multi-pathway inhibition strategy - Targeting invasion-related signaling in HCC	Yen et al. (2015)
*In vivo:* NDEA-induced HCC in rats	- Prevents tumor initiation and progression - Antioxidant and hepatoprotective effects	- Xenobiotic-metabolizing enzymes (XMEs) modulation - Lipid peroxidation ↓ - Antioxidant system ↑	- Reduced preneoplastic lesions - Decreased cytochrome P450 activity - Increased GST activity and antioxidant status	- Chemopreventive agent against carcinogen-induced HCC - Protects liver from oxidative and chemical damage	Arul and Subramanian (2013)
*In vitro:* Hepa-1c1c7 cells	- Inhibits carcinogen-activating enzyme expression	- CYP1A1 ↓ - Aryl hydrocarbon receptor (AhR) ↓ - DRE binding inhibition	- Reduced TCDD-induced CYP1A1 mRNA and enzyme activity - Inhibition of AhR-DNA binding	- Blocks carcinogen activation pathways - Potential role in cancer prevention - Modulates xenobiotic metabolism	Kim et al. (2004)

Abbreviations: HCC, hepatocellular carcinoma; HUVECs, human umbilical vein endothelial cells; CAM, chorioallantoic membrane; VEGFA, vascular endothelial growth factor A; c-Met, mesenchymal–epithelial transition factor; PD-L1, programmed death-ligand 1; CD8^+^, cluster of differentiation eight positive T cells; CCK-8, Cell Counting Kit-8; WB, western blot; qPCR, quantitative polymerase chain reaction; IGFBP3, insulin-like growth factor binding protein 3; PGF, placental growth factor; CA9, carbonic anhydrase IX; AKR1C3, aldo-keto reductase family one member C3; KLK1, kallikrein 1; CHRNA7, cholinergic receptor nicotinic alpha seven subunit; NPs, nanoparticles; NARNPs, naringenin nanoparticles; DOX, doxorubicin; ATG5, autophagy-related gene 5; LC3, microtubule-associated protein one light chain 3; IBU, ibuprofen; CI, combination index; AMPK, AMP-activated protein kinase; PGC1α, peroxisome proliferator-activated receptor gamma coactivator 1-alpha; ROS, reactive oxygen species; JAK2, Janus kinase 2; STAT3, signal transducer and activator of transcription 3; ΔΨm, mitochondrial membrane potential; EMT, epithelial–mesenchymal transition; HIF-1, hypoxia-inducible factor 1; GSK3β, glycogen synthase kinase three beta; DEN, diethylnitrosamine; 2AAF, 2-acetylaminofluorene; ALT, alanine aminotransferase; AST, aspartate aminotransferase; ALP, alkaline phosphatase; γ-GT, gamma-glutamyl transferase; AFP, alpha-fetoprotein; CEA, carcinoembryonic antigen; CA19.9, carbohydrate antigen 19-9; NO, nitric oxide; SOD, superoxide dismutase; GPx, glutathione peroxidase; CAT, catalase; GSH, glutathione; NF-κB, nuclear factor kappa B; AP-1, activator protein 1; ERK, extracellular signal-regulated kinase; JNK, c-Jun N-terminal kinase; PI3K, phosphatidylinositol 3-kinase; NDEA, N-nitrosodiethylamine; GST, glutathione S-transferase; XMEs, xenobiotic-metabolizing enzymes; CYP1A1, cytochrome P450 1A1; AhR, aryl hydrocarbon receptor; DRE, dioxin response element.

*In vitro* studies showed that NAR nanoparticles significantly reduce IC50 values in HepG2 cells compared with free NAR. The dominant mechanism is enhanced intracellular delivery leading to stronger inhibition of tumor-cell viability. Secondary mechanisms include improved solubility, stability, and bioavailability, resulting in enhanced therapeutic efficiency relative to free NAR (Elnawasany et al., 2023). *In vitro* evidence indicates that NAR induces apoptosis in HepG2 cells. The dominant mechanism is ROS-mediated mitochondrial apoptosis involving caspase-3 and caspase-9 activation. Secondary mechanisms include inhibition of JAK2/STAT3 survival signaling, leading to reduced cell viability (Zhang et al., 2023). *In vitro* findings show dose-dependent synergism between NAR and tamoxifen. The dominant mechanism is ROS-mediated p53 activation and mitochondrial dysfunction. Secondary mechanisms include suppression of proliferation, migration, and invasion, resulting in enhanced anticancer sensitivity (Xu et al., 2022). *In vitro* studies demonstrate that NAR suppresses HCC invasion. The dominant mechanism is inhibition of matrix metalloproteinase-9 (MMP-9) via AP-1 and NF-κB signaling pathways. Secondary mechanisms include suppression of extracellular signal-regulated kinase/c-Jun N-terminal kinase (ERK/JNK) and PI3K/Akt pathways, leading to reduced metastatic potential (Yen et al., 2015). *In vitro* evidence shows that NAR inhibits cytochrome P450 family one subfamily A member 1 (CYP1A1) expression. The dominant mechanism is blockade of aryl hydrocarbon receptor binding to dioxin response elements, thereby suppressing xenobiotic activation and carcinogen metabolism (Kim et al., 2004).

### 4.2 *In vitro* and *in vivo* evidence (translational studies)

Both *in vitro* and *in vivo* evidence demonstrate strong anticancer and immunomodulatory effects of NAR, with higher translational relevance observed *in vivo*. The dominant mechanism is K48-linked degradation of VEGFA and c-Met, leading to suppression of angiogenesis and PD-L1 expression. Secondary mechanisms include activation of CD8^+^ T-cell–mediated antitumor immunity. These molecular effects result in reduced tumor growth, angiogenesis, and immune evasion. Combination therapy with PD-L1 antibodies or bevacizumab further enhances efficacy, highlighting strong translational potential (Wu W. et al., 2025). Both *in vitro* and *in vivo* studies show that NAR enhances ferroptosis-based therapy. The dominant mechanism is AMPK–peroxisome proliferator-activated receptor gamma coactivator 1-alpha (PGC1α) activation, leading to inhibition of glycolysis and increased ferroptosis sensitivity. Secondary mechanisms include enhanced lipid peroxidation. These effects promote ferroptotic cell death and tumor suppression (Li et al., 2024). *In vitro* and *in vivo* evidence show that 6-CEPN inhibits HCC progression. The dominant mechanism is inhibition of Wnt/β-catenin signaling via β-catenin degradation and GSK3β activation. Secondary mechanisms include suppression of HIF-1 signaling and cancer stemness pathways. These molecular events reduce EMT, metastasis, and tumor growth *in vivo*, indicating strong translational relevance (Kang et al., 2019).

### 4.3 *In vivo* evidence

*In vivo* DEN-induced HCC model showed that NAR combined with quercetin improves liver pathology. The dominant mechanism is enhancement of antioxidant defense systems such as SOD, catalase (CAT), glutathione peroxidase (GPx), and GSH, reducing oxidative stress. Secondary mechanisms include modulation of inflammatory and apoptotic signaling, leading to reduced tumor markers and improved hepatic function (Ahmed et al., 2022). *In vivo* NDEA model demonstrated chemopreventive effects of NAR. The dominant mechanism is restoration of antioxidant defense and xenobiotic metabolism. This reduces lipid peroxidation and improves liver function, thereby suppressing hepatocarcinogenesis (Arul and Subramanian, 2013).

### 4.4 Mechanistic integration and pharmacokinetics

Overall, NAR exerts anticancer effects through a hierarchical and integrated network of mechanisms. The dominant pathways include inhibition of Wnt/MAPK signaling, suppression of NF-κB/angiogenesis axis, activation of mitochondrial apoptosis, metabolic reprogramming via AMPK signaling, and regulation of xenobiotic metabolism. Secondary mechanisms include ROS modulation, immune activation, autophagy induction, and detoxification-related effects.

These molecular events translate into four major biological outcomes.

1. Induction of apoptosis and ferroptosis,
2. Inhibition of proliferation, migration, and metastasis,
3. Suppression of angiogenesis and immune evasion, and
4. Restoration of metabolic and redox homeostasis.

Importantly, poor aqueous solubility and low systemic bioavailability limit the clinical efficacy of free NAR, whereas nanoparticle-based delivery systems significantly enhance pharmacokinetic properties, including solubility, stability, controlled release, circulation time, and tumor-targeted accumulation. Consequently, nanoformulated NAR exhibits superior bioavailability and markedly improved anticancer efficacy compared with free NAR, supporting its strong translational potential for HCC therapy.

### 4.5 Critical analysis, limitations, and translational perspectives

Collectively, the body of research indicates that NAR manifests a diverse array of anticancer effects in HCC through the modulation of apoptosis, ferroptosis, oxidative stress, angiogenesis, inflammatory responses, and various signaling cascades, including Wnt/β-catenin, MAPK, AMPK/PGC1α, JAK2/STAT3, and PI3K/Akt. Nevertheless, the extant evidence remains heterogeneous and is accompanied by numerous limitations. The majority of existing studies predominantly rely on *in vitro* methodologies utilizing a restricted selection of HCC cell lines, while comparatively fewer investigations have corroborated these findings in *vivo* animal models or translational contexts. Furthermore, significant variability is observed concerning NAR dosage, formulation, treatment duration, and combinatorial therapeutic approaches, which complicates direct comparisons among studies. Importantly, nanoparticle-based drug delivery systems have consistently exhibited superior antiproliferative efficacy and enhanced bioavailability relative to free NAR formulations, indicating a potential for improved therapeutic outcomes. Moreover, disparate findings were identified in ROS-related mechanisms, wherein NAR was observed to stimulate ROS-mediated apoptosis and ferroptosis in specific studies, while concurrently demonstrating antioxidant and cytoprotective effects in others, contingent upon concentration and experimental conditions. In a similar vein, combination therapies exhibited outcomes that were dependent on the context, as high-dose flavonoid combinations augmented anticancer efficacy, whereas reduced concentrations sporadically diminished cytotoxic responses. Consequently, although the extant preclinical data endorses the potential therapeutic function of NAR in HCC, further rigorously designed *in vivo* studies, pharmacokinetic assessments, and clinical validations are imperative to ascertain its translational relevance and therapeutic dependability. Nanoencapsulation strategies consistently enhanced NAR cytotoxicity, cellular uptake, and pharmacokinetic performance compared with free NAR formulations.

## 5 Molecular targets and signaling pathways modulated by HSD in HCC

HSD has demonstrated multi-level anticancer activity in HCC, with evidence spanning *in vitro* cellular models and *in vivo* experimental hepatocarcinogenesis models. Importantly, the overall evidence suggests that while *in vitro* findings dominate mechanistic resolution, *in vivo* studies provide stronger translational relevance, particularly in terms of tumor suppression, chemoprevention, and liver protection.

### 5.1 *In vitro* evidence

HSD, diosmin, and cisplatin reduced HepG2 viability with synergistic cytotoxicity (CI < 1), inducing apoptosis and G2/M arrest while sparing normal Vero cells, where the dominant mechanism involves mitochondrial apoptosis and cell-cycle arrest via Cyclin D1 suppression, and secondary mechanisms include senescence modulation and reduced cisplatin-induced toxicity in normal cells, ultimately leading to decreased tumor cell survival *in vitro* (Artanti et al., 2024) (Table 2). Cyclopia extracts and HSD reduced cancer cell viability mainly through ATP depletion and mitochondrial disruption while inducing autophagy without apoptosis, where the dominant mechanism is metabolic collapse via ATP depletion and secondary mechanisms include autophagy induction and iron–polyphenol-mediated mitochondrial interactions, resulting in non-apoptotic cytostatic growth inhibition (Samodien et al., 2024). HSD-rich citrus extract showed stronger anticancer effects than isolated compounds by inducing apoptosis and reducing invasion, where the dominant mechanism is synergistic multi-compound apoptosis activation and secondary mechanisms involve suppression of invasion-related pathways, leading to enhanced tumor suppression (Phucharoenrak et al., 2023). HSD bound calmodulin-dependent protein kinase IV (CAMKIV) and inhibited proliferation via mitochondrial apoptosis through caspase-3 activation and Bax upregulation, where the dominant mechanism is CAMKIV inhibition leading to intrinsic apoptotic signaling and secondary mechanisms include disruption of calcium-dependent proliferative signaling, resulting in target-driven apoptosis (Naz et al., 2019).

**TABLE 2:** Effects and molecular mechanisms of hesperidin in hepatocellular carcinoma.

Model/System	Main effects	Molecular targets/Pathways	Key findings	Therapeutic implications	Study
*In vitro:* HepG2 (cancer), Vero (normal kidney) cells	- Enhances cytotoxicity of cisplatin - Induces apoptosis - Modulates cellular senescence	- Apoptosis pathways (Annexin V/PI) - Senescence-associated β-gal (SA-β-gal)	- Synergistic effect (CI < 1) with cisplatin - Increased apoptosis in HepG2 cells - Reduced senescence in normal kidney cells - Selective cytotoxicity toward cancer cells	- Promising adjuvant to chemotherapy - Reduces cisplatin-induced toxicity in normal cells - Enhances therapeutic index	Artanti et al. (2024)
*In vitro:* HepG2, HT-29 cells	- Limited direct anticancer effect - No apoptosis induction - Modulates autophagy (indirectly via extracts)	- Autophagy pathways - Mitochondrial integrity disruption (indirect)	- Hesperidin alone showed no significant cytotoxic effect - Extracts reduced viability and proliferation - Effects likely due to synergistic polyphenols rather than hesperidin alone	- Highlights importance of phytochemical synergy - Suggests hesperidin may act better in combination rather than alone	Samodien et al. (2024)
*In vitro:* PLC/PRF/5 liver cancer cells	- Induces apoptosis - Inhibits invasion - Antiproliferative effect	- Apoptosis pathways (Annexin-PI) - Likely multi-target phytochemical interactions	- Extract more potent than pure hesperidin or limonin alone - Synergistic effect between hesperidin and limonin - Strong inhibition of invasion and enhanced apoptosis	- Supports use of whole extracts over isolated compounds - Potential nutraceutical or phytochemical-based therapy	Phucharoenrak et al. (2023)
*In vitro:* HepG2, SH-SY5Y cells	- Inhibits proliferation - Induces apoptosis - Mitochondrial dysfunction	- CAMKIV inhibition - Bax ↑ - Caspase-3 activation ↑ - Mitochondrial membrane potential (ΔΨm) ↓	- Strong binding affinity to CAMKIV - Activation of intrinsic apoptotic pathway - Reduced cell viability and proliferation	- Novel molecular target (CAMKIV) - Potential targeted therapy approach - Broad anticancer applicability	Naz et al. (2019)
*In vivo:* DEN-induced HCC in rats	- Inhibits tumor development - Antioxidant and hepatoprotective effects	- PI3K ↓ - Akt ↓ - CDK-2 ↓	- Reduced liver enzymes and AFP levels - Suppressed oxidative stress - Preserved liver histological integrity	- Targets oncogenic PI3K/Akt signaling - Chemopreventive and therapeutic potential in HCC	Mo’men et al. (2019)
*In vivo:* DEN/CCl_4_-induced HCC in rats	- Anti-inflammatory and antioxidant effects - Inhibits proliferation and fibrosis - Prevents hepatocarcinogenesis	- Nrf2/ARE/HO-1 ↑ - PPARγ ↑ - NF-κB ↓ - TGF-β1/Smad3 ↓	- Reduced tumor markers and liver enzymes - Decreased lipid peroxidation and collagen deposition - Suppressed NF-κB and TGF-β1 signaling	- Multi-pathway chemopreventive agent - Targets oxidative stress, inflammation, and fibrosis - Promising for preventing HCC progression	Mahmoud et al. (2017)
*In vivo:* TAA-induced HCC in rats *In vitro:* HepG2 cells	- Hepatoprotective effect - Inhibits tumor progression - Anti-inflammatory and antioxidant effects	- Wnt3a/β-catenin ↓ - Wnt5a ↓ - Cyclin D1 ↓ - Caspase-3 ↑	- Suppressed canonical and non-canonical Wnt pathways - Improved liver function and oxidative status - Reduced proliferation markers	- Targets Wnt signaling in HCC - Prevents early hepatocarcinogenesis - Promising prophylactic agent	Zaghloul et al. (2017)
*In vitro:* HL60 cells *In vivo:* DEN-induced HCC rat model	- Cytotoxic effect - Epigenetic modulation (hypomethylation) - Chemopreventive activity	- DNA methylation ↓ (LINE-1, ALU sequences)	- Dose-dependent cytotoxicity - Significant hypomethylation of repetitive DNA sequences - Reduced tumor nodules *in vivo*	- Potential epigenetic therapy agent - Modulates DNA methylation in cancer - Chemopreventive application	Fernández-Bedmar et al. (2017)
*In vitro:* HepG2 cells	- Induces paraptosis (non-apoptotic cell death) - Mitochondrial dysfunction - ROS generation	- Ca2+ signaling ↑ (ER → mitochondria) - IP3R, ryanodine receptor ↑ - ROS ↑ - Mitochondrial membrane disruption	- Mitochondrial Ca2+ overload triggers cell death - ER-mediated Ca2+ release essential for effect - ROS acts downstream of Ca2+ signaling	- Novel mechanism: paraptosis induction - Alternative to apoptosis-based therapies - Targets mitochondrial and ER stress pathways	Yumnam et al. (2016)
*In vitro:* HepG2 cells	- Induces apoptosis - Mitochondrial dysfunction - Activates extrinsic and intrinsic pathways	- Caspase-3, -8, -9 ↑ - Bax, Bak, tBid ↑ - Bcl-xL ↓ - ΔΨm ↓	- Dose-dependent apoptosis induction - Loss of mitochondrial membrane potential - No ROS involvement - Activation of both intrinsic and extrinsic apoptotic pathways	- Strong pro-apoptotic agent - Dual-pathway apoptosis induction - Potential anticancer drug candidate	Banjerdpongchai et al. (2016)
*In vitro:* HepG2 cells	- Anti-invasive and anti-metastatic effects - Reduces MMP-9 activity	- NF-κB ↓ - AP-1 ↓ - p38/JNK ↓ - IκB signaling ↑	- Suppressed MMP-9 transcription and secretion - Inhibited NF-κB nuclear translocation - Reduced TPA-induced invasion	- Potent anti-metastatic agent - Targets invasion-related signaling pathways	Lee et al. (2010)
*In vitro:* HepG2 cells	- Anti-invasive effect - Inhibits metastasis-related gene expression	- NF-κB ↓ - AP-1 ↓ - p38/JNK ↓ - MMP-9 ↓	- Suppressed acetaldehyde-induced MMP-9 expression and secretion - Inhibited NF-κB and AP-1 signaling pathways - Reduced cancer cell invasiveness	- Potential therapy for alcohol-related HCC - Anti-metastatic and chemopreventive agent	Yeh et al. (2009)

Abbreviations: HCC, hepatocellular carcinoma; HSD/HES, hesperidin; DSM, diosmin; Cisp, cisplatin; HepG2, human hepatocellular carcinoma cell line; Vero, African green monkey kidney cells; IC50, half maximal inhibitory concentration; CI, combination index; SI, selectivity index; SA-β-gal, senescence-associated β-galactosidase; CAMKIV, calcium/calmodulin-dependent protein kinase IV; bax, Bcl-2-associated X protein; ΔΨm, mitochondrial membrane potential; PI3K, phosphatidylinositol 3-kinase; Akt, protein kinase B; CDK-2, cyclin-dependent kinase 2; DEN, diethylnitrosamine; CCl_4_, carbon tetrachloride; AFP, alpha-fetoprotein; Nrf2, nuclear factor erythroid 2-related factor 2; ARE, antioxidant response element; HO-1, heme oxygenase-1; PPARγ, peroxisome proliferator-activated receptor gamma; NF-κB, nuclear factor kappa B; TGF-β1, transforming growth factor beta 1; Smad3, mothers against decapentaplegic homolog 3; PCNA, proliferating cell nuclear antigen; Wnt, wingless-related integration site; β-catenin, beta-catenin; IP3R, inositol 1,4,5-triphosphate receptor; JNK, c-Jun N-terminal kinase; AP-1, activator protein 1; MMP-9, matrix metalloproteinase-9.

HSD induced apoptosis via intrinsic and extrinsic pathways with caspase-8/9/3 activation and Bcl-xL downregulation, where the dominant mechanism is dual apoptotic pathway activation and secondary mechanisms include mitochondrial depolarization independent of ROS, leading to programmed cell death (Banjerdpongchai et al., 2016). HSD suppressed TPA-induced invasion by inhibiting NF-κB and AP-1 via p38/JNK pathways, where the dominant mechanism is transcriptional repression of MMP-9 and secondary mechanisms include MAPK inhibition, resulting in reduced metastatic potential (Lee et al., 2010). HSD also blocked acetaldehyde-induced MMP-9 expression via NF-κB/AP-1 inhibition, where the dominant mechanism is transcriptional blockade of metastatic signaling and secondary mechanisms involve inflammatory pathway suppression, leading to decreased invasion (Yeh et al., 2009).

### 5.2 *In vivo* evidence (higher translational relevance)

In DEN-induced HCC rats, HSD reduced AFP and liver enzymes while inhibiting PI3K/Akt and CDK2 signaling, where the dominant mechanism is suppression of survival and cell-cycle pathways and secondary mechanisms include oxidative stress reduction, demonstrating strong chemopreventive translational relevance *in vivo* (Mo’men et al., 2019). HSD attenuated DEN/CCl4-induced hepatocarcinogenesis by inhibiting NF-κB and transforming growth factor-beta/SMAD family member 3 (TGF-β/Smad3) signaling while activating Nrf2/HO-1 pathways, where the dominant mechanism is suppression of inflammatory and fibrotic signaling and secondary mechanisms include activation of antioxidant defenses, resulting in reduced fibrosis and oxidative damage (Mahmoud et al., 2017). HSD inhibited Wnt3a/β-catenin signaling *in vivo* and *in vitro*, reducing Cyclin D1 and restoring oxidative balance, where the dominant mechanism is Wnt pathway inhibition and secondary mechanisms include redox homeostasis restoration, leading to reduced proliferation and tumor progression (Zaghloul et al., 2017). HSD induced DNA hypomethylation and reduced hepatic nodules in DEN-induced HCC rats, where the dominant mechanism is epigenetic reprogramming via LINE-1/ALU hypomethylation and secondary mechanisms include suppression of early tumor initiation, resulting in chemopreventive effects (Fernández-Bedmar et al., 2017). HSD induced paraptosis via mitochondrial Ca^2+^ overload from ER release through IP3R and ryanodine receptors, where the dominant mechanism is Ca^2+^-mediated mitochondrial dysfunction and secondary mechanisms include ROS generation downstream of mitochondrial stress, resulting in non-apoptotic cell death (Yumnam et al., 2016).

### 5.3 Mechanistic integration and pharmacokinetics

**FIGURE 2:**
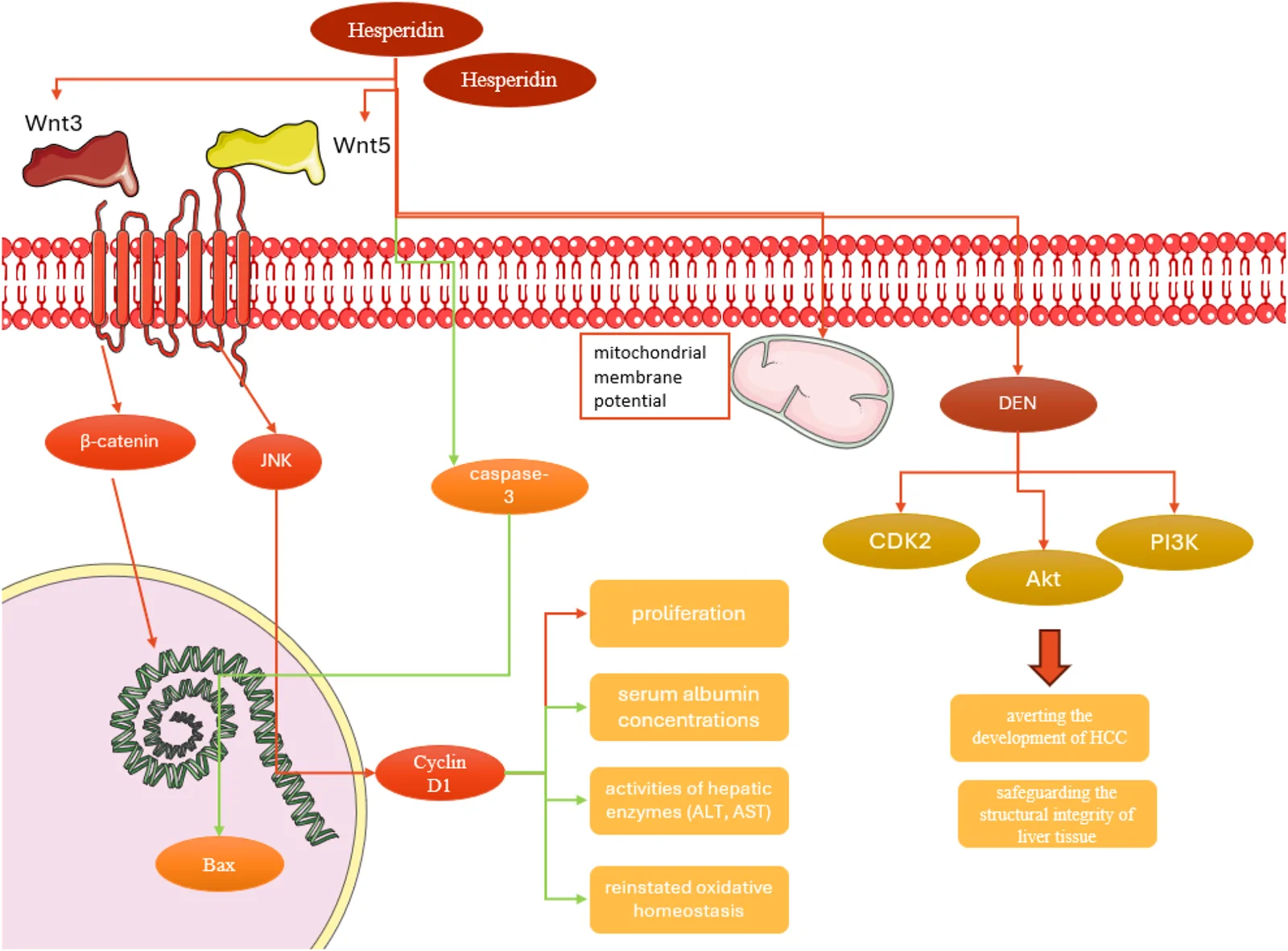
Molecular signaling pathways modulated by hesperidin in hepatocellular carcinoma (HCC).

Across studies, HSD exerts anticancer effects through hierarchically organized signaling networks, where dominant pathways include mitochondrial apoptosis (caspase cascade), PI3K/Akt inhibition, Wnt/β-catenin suppression, and NF-κB/AP-1-mediated anti-metastatic signaling, while secondary mechanisms include oxidative stress modulation, epigenetic reprogramming, Ca^2+^-dependent paraptosis, autophagy induction, and inflammatory microenvironment regulation, collectively converging into biological outcomes such as induction of apoptotic and non-apoptotic cell death, inhibition of proliferation and invasion, suppression of metastasis, and restoration of redox and metabolic homeostasis. Importantly, *in vivo* models (DEN, CCl_4_, TAA) provide the strongest translational evidence compared with *in vitro* systems, demonstrating consistent tumor suppression and hepatoprotection, while *in vitro* studies primarily elucidate molecular mechanisms. From a pharmacokinetic perspective, free HSD is limited by poor bioavailability and systemic exposure, whereas extract-based and formulation strategies improve stability, solubility, and cellular uptake through synergistic polyphenol interactions, indicating that delivery optimization is a critical determinant of translational efficacy in HCC therapy (Figure 2).

### 5.4 Critical analysis, limitations, and translational perspectives

The cumulative body of evidence indicates that HSD exerts a complex array of anticancer effects in HCC through the modulation of apoptosis, oxidative stress, inflammation, metastasis, epigenetic regulation, and various signaling pathways, including PI3K/Akt, NF-κB, Wnt/β-catenin, TGF-β/Smad3, and CAMKIV, and MAPK-related cascades. Numerous studies have consistently shown that HSD inhibits cellular proliferation, migration, invasion, and metastatic characteristics while promoting apoptotic or paraptotic cell death in models of HCC. Additionally, combinatorial approaches that incorporate HSD with cisplatin or complex phytochemical extracts frequently yield synergistic anticancer effects, thereby underscoring the potential therapeutic significance of multi-targeted interventions. Nonetheless, notable limitations and inconsistencies persist within the existing literature. The majority of investigations have primarily utilized *in vitro* HepG2-based models, while comparatively few studies have corroborated these findings in animal models or translational contexts, thereby constraining direct clinical relevance. There also exists considerable heterogeneity concerning HSD concentrations, treatment durations, experimental conditions, and formulation strategies, which complicates direct comparisons among studies. Although nanoformulation studies on HSD remain limited, available evidence suggests that delivery engineering may improve intracellular uptake, stability, and therapeutic persistence. Moreover, numerous investigations have assessed HSD within the context of intricate plant extracts that comprise various bioactive constituents, thereby complicating the attribution of the observed anticancer effects exclusively to HSD or to synergistic interactions among phytochemicals. Importantly, mechanistic variations were noted in relation to the modulation of ROS and the pathways leading to cellular demise. While certain studies have indicated that apoptosis and mitochondrial dysfunction are contingent upon ROS, others have identified ROS-independent apoptotic pathways or alternative modalities of programmed cell death, including paraptosis. In a similar vein, the documented anticancer effects were found to be contingent upon the cellular environment, signaling milieu, and co-administered therapeutic agents. Research utilizing chemically induced HCC animal models along with pathway-specific mechanistic investigations typically furnish more robust translational evidence in comparison to isolated cytotoxicity assays. Nonetheless, the lack of clinical trials, pharmacokinetic assessments, and standardized dosing protocols continues to represent a significant obstacle to the therapeutic application of HSD in the management of HCC. Therefore, additional well-designed *in vivo* investigations and clinical validations are necessary to clarify the long-term efficacy, safety, bioavailability, and mechanistic specificity of HSD-based therapeutic approaches in HCC.

Schematic representation of the intracellular signaling networks through which hesperidin is proposed to modulate key pathways involved in hepatocellular carcinoma progression. Hesperidin is associated with modulation of Wnt signaling through regulation of Wnt3/Wnt5-related receptor activity, which may contribute to attenuation of β-catenin–dependent transcriptional signaling. It is also suggested to influence MAPK signaling, particularly JNK-associated pathways. Within cellular systems, hesperidin is linked to the induction of apoptotic processes through caspase-3 activation, disruption of mitochondrial membrane potential, and modulation of pro-apoptotic proteins such as Bax, which collectively may contribute to reduced cell proliferation and downregulation of cell-cycle regulators including cyclin D1. In addition, hesperidin is associated with modulation of PI3K/Akt and CDK2 signaling pathways, which may contribute to decreased tumor cell survival and proliferation. These molecular interactions are further linked to potential hepatoprotective effects, including restoration of oxidative homeostasis and normalization of hepatic biochemical markers (ALT and AST), thereby supporting maintenance of liver tissue integrity. Overall, the diagram should be interpreted as an integrative conceptual model summarizing the most consistently reported experimental observations, rather than a definitive mechanistic pathway, as the reported effects may vary depending on experimental context and model system.

## 6 Molecular targets and signaling pathways modulated by HST in HCC

### 6.1 *In vitro* evidence

Evidence from an *in vitro* study demonstrated that HST selectively reduced the viability of HepG2 HCC cells while exerting minimal toxicity toward NIH-3T3 normal fibroblasts, suggesting potential therapeutic selectivity. Co-treatment with cisplatin (Cisp) produced synergistic cytotoxicity (CI < 1), indicating possible translational value as an adjuvant therapy. Mechanistically, the dominant pathway involved inhibition of the ERK1/2–Cyclin D1 signaling axis together with activation of mitochondrial apoptosis, evidenced by increased Bax and caspase-3 and reduced Bcl-xL, p-ERK1/2, and Cyclin D1. Secondary effects included enhanced senescence and reduced colony formation. These molecular alterations resulted in G2/M arrest, increased apoptosis, and suppression of HCC-cell proliferation, whereas HST attenuated cisplatin-induced senescence in normal fibroblasts (Artanti et al., 2026) (Table 3). An *in vitro* HepG2 study showed that HSD and apigenin enhanced doxorubicin cytotoxicity, suggesting their potential use as metabolic sensitizers in HCC therapy. The dominant mechanism was suppression of glycolytic metabolism through downregulation of HK2 and LDHA, thereby inhibiting the Warburg effect. In contrast, ROS generation and oxidative DNA damage represented secondary mechanisms, since synergistic cytotoxicity occurred independently of oxidative stress. Mechanistically, inhibition of tumor energy metabolism increased antiproliferative activity and promoted tumor-cell death (Korga et al., 2019).

**TABLE 3:** Effects and molecular mechanisms of hesperetin in hepatocellular carcinoma.

Compound/Formulation	Model/Cell line	Key methods	Key findings	Study
Hesperetin (HST) + Cisplatin (Cisp)	HepG2 (HCC), NIH-3T3 fibroblasts	MTT assay, colony formation, SA-β-gal staining, flow cytometry, Western blot	Synergistic reduction in HepG2 viability, increased apoptosis and G2/M arrest, protection of normal fibroblasts from senescence	Artanti et al. (2026)
Hesperetin (HST)	HepG2 (HCC) *in vitro* and xenograft *in vivo*	MTT assay, Hoechst 33,258 staining, Western blot, ROS/ATP/Ca^2+^ measurement	Induced apoptosis via mitochondrial pathway, increased ROS, ATP, Ca^2+^, upregulated cytosolic AIF, Apaf-1, Cyt C, caspase-3/-9, Bax, downregulated Bcl-2	Zhang et al. (2015)
Hesperetin-conjugated PEGylated gold nanoparticles (Au-mPEG (5000)-S-HP NPs)	Male Wistar rats, DEN-induced hepatocarcinogenesis	HR-TEM + EDAX, DLS, Zeta potential, PBS release, histology, TNF-α, NF-κB, PCNA, glycoconjugates, mast cell counts	Suppressed inflammation, proliferation, and tumor progression in DEN-induced liver cancer; improved antioxidant and anti-proliferative effects	Krishnan et al. (2017)
Hesperetin-conjugated PEGylated gold nanoparticles (Au-mPEG (5000)-S-HP)	Male Wistar rats, DEN-induced HCC	Dose-fixation, toxicity, body/liver weight, nodule analysis, tumor markers (CEA, AFP), lipid peroxidation, antioxidant enzyme assays, histology	Au-mPEG (5000)-S-HP improved bioavailability and targeting, reduced hepatic damage, lipid peroxidation, and restored antioxidant enzymes more effectively than pure HP	Gokuladhas et al. (2016)
Hesperidin + Sesame extract (MSO)	Rats, DEN-induced hepatocarcinogenesis	GST-P-positive foci counting, PCNA staining, apoptosis assays, hepatic triglycerides, fatty acid synthase, cytochrome P450	MSO enhanced chemopreventive effect of hesperidin in early HCC by reducing preneoplastic foci, increasing apoptosis, decreasing proliferation, and modulating hepatic lipogenesis	Khuanphram et al. (2021)
Hesperetin + Apigenin, Doxorubicin (DOX)	HepG2 (human HCC)	Cell viability, ROS measurement, DNA oxidative damage, lipid peroxidation, double-strand breaks, mRNA expression of glycolytic genes (HK2, LDHA)	Hesperetin and apigenin augmented doxorubicin cytotoxicity via modulation of glycolytic genes and increased chemosensitivity, independent of oxidative stress	Korga et al. (2019)

Abbreviations: HCC: hepatocellular carcinoma; Cisp: cisplatin; HST: hesperetin; ROS: reactive oxygen species; ATP: adenosine triphosphate; Ca^2+^: calcium; Au-mPEG (5000)-S-HP: hesperetin-conjugated PEGylated gold nanoparticles; DEN: diethylnitrosamine; HP: hesperetin; MSO: mixed sesame and orange seed extract; PCNA: proliferating cell nuclear antigen; GST-P: glutathione S-transferase P; DOX: doxorubicin; HK2: hexokinase 2; LDHA: lactate dehydrogenase A; SOD: superoxide dismutase; CAT: catalase; GSH: glutathione; GPx: glutathione peroxidase; GR: glutathione reductase.

### 6.2 Combined *in vitro* and *in vivo* evidence

Both *in vitro* and *in vivo* studies supported the anticancer activity of HST against HCC, although xenograft findings provide greater translational relevance. The dominant mechanism involved activation of mitochondrial apoptosis through increased AIF, Apaf-1, cytochrome c, caspase-9, caspase-3, and Bax together with suppression of Bcl-2. Secondary mechanisms included ROS modulation, ATP depletion, and Ca^2+^ imbalance, contributing to mitochondrial dysfunction. These molecular changes promoted apoptosis and significantly suppressed tumor growth in xenograft models. Overall, HST induced mitochondrial dysfunction and caspase activation, leading to apoptotic elimination of HCC cells and inhibition of tumor progression (Zhang et al., 2015).

### 6.3 *In vivo* evidence

Evidence from an *in vivo* DEN-induced hepatocarcinogenesis model demonstrated that HST-loaded gold nanoparticles (Au-mPEG (5000)-S-HST NPs) exerted stronger anticancer effects than free HST, highlighting the translational importance of nanoparticle delivery. The dominant pathway involved suppression of NF-κB inflammatory signaling, evidenced by reduced tumor necrosis factor-alpha (TNF-α) expression, NF-κB activation, and mast-cell infiltration. Secondary effects included reduced PCNA expression and inhibition of tumor-cell proliferation. Importantly, nanoparticle formulation improved drug stability, release profile, and bioavailability compared with free HST, resulting in enhanced suppression of hepatocarcinogenesis (Krishnan et al., 2017). An *in vivo* DEN-induced HCC model demonstrated chemopreventive effects of both free HST and pegylated gold nanoparticle-conjugated HST, although nanoparticle delivery showed greater translational potential because of improved pharmacokinetics and bioavailability. The dominant mechanism involved attenuation of oxidative stress through restoration of antioxidant defenses, including SOD, CAT, GSH, GPx, and glutathione reductase, together with reduced lipid peroxidation. Secondary mechanisms included anti-inflammatory and antiproliferative effects associated with reductions in tumor biomarkers and hepatic injury markers. Compared with free HST, nanoparticle conjugation improved solubility, stability, and targeted delivery, leading to stronger hepatoprotection and inhibition of HCC progression (Gokuladhas et al., 2016).

**FIGURE 3:**
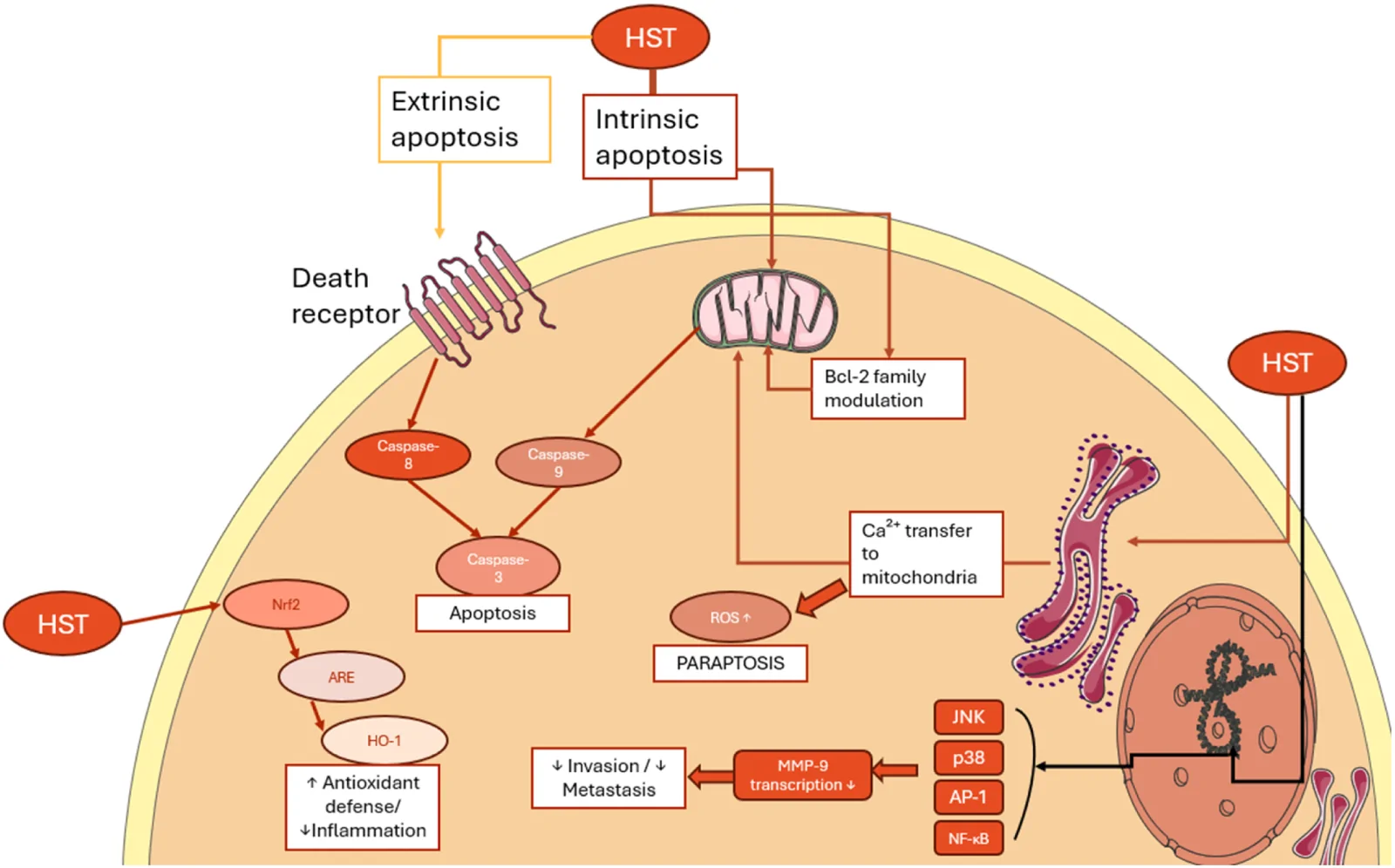
Molecular targets and signaling pathways modulated by HST in hepatocellular carcinoma (HCC).

Evidence from an *in vivo* DEN-induced hepatocarcinogenesis model showed that combined HSD and sesamin treatment exerted greater chemopreventive activity than HSD alone. The dominant mechanisms involved suppression of proliferation and induction of apoptosis, evidenced by reduced GST-P-positive foci and PCNA-positive hepatocytes together with increased apoptotic cell counts. A secondary mechanism included modulation of lipid metabolism through downregulation of FASN and reduced hepatic triglyceride accumulation. These molecular alterations were associated with attenuation of early hepatocarcinogenesis and suppression of tumor progression. Moreover, minimal effects on CYP450 enzymes suggest favorable translational potential and safety (Khuanphram et al., 2021). Collectively, these findings suggest that HST and related flavonoids suppress HCC progression primarily through modulation of mitochondrial apoptosis, inflammatory signaling, oxidative stress, and metabolic reprogramming, ultimately leading to reduced proliferation and enhanced tumor-cell death (Figure 3).

Schematic representation of the intracellular signaling networks through which HST is proposed to exert antitumor and hepatoprotective effects in hepatocellular carcinoma. HST is associated with activation of both extrinsic and intrinsic apoptotic pathways. In the extrinsic pathway, modulation of death receptor signaling may contribute to caspase-8 activation and subsequent caspase-3-mediated apoptosis. In the intrinsic mitochondrial pathway, HST appears to regulate Bcl-2 family proteins, leading to mitochondrial dysfunction, caspase-9 activation, and apoptotic cell death. HST is also linked to induction of paraptosis through ER-associated Ca^2+^ transfer to mitochondria, resulting in mitochondrial Ca^2+^ overload, ROS accumulation, and mitochondrial stress. In addition, HST may suppress tumor invasion and metastatic behavior through inhibition of NF-κB-, AP-1-, p38-, and JNK-associated signaling pathways, thereby reducing MMP-9 transcription and activity. Furthermore, HST is associated with activation of antioxidant and anti-inflammatory responses through modulation of the Nrf2/ARE/HO-1 signaling axis. Collectively, these interconnected molecular events may contribute to inhibition of tumor growth and metastasis while supporting hepatoprotective effects and restoration of redox homeostasis. Overall, the diagram should be interpreted as an integrative conceptual summary of currently available experimental evidence rather than a fully established mechanistic pathway, as the reported effects may vary depending on experimental model and biological context.

### 6.4 Mechanistic integration and pharmacokinetics

Overall, HST and related natural compounds exert anticancer effects in HCC through a multi-target and hierarchically organized network of mechanisms, in which the dominant pathways include activation of mitochondrial-mediated apoptosis (via Bax, caspase-3/9, cytochrome c, and Apaf-1), suppression of NF-κB and inflammatory signaling, inhibition of cell-cycle progression (Cyclin D1 and G2/M arrest), and modulation of metabolic reprogramming including glycolysis and lipogenesis (e.g., HK2, LDHA, and FASN). Secondary mechanisms include regulation of oxidative stress balance (ROS, SOD, CAT, GSH, GPx), attenuation of angiogenic and proliferative signaling, and modulation of energy homeostasis and xenobiotic metabolism. These molecular events collectively converge into key biological outcomes, namely, induction of apoptosis and suppression of tumor cell proliferation, reduction of preneoplastic lesion formation and tumor progression, inhibition of metastasis-related metabolic reprogramming, and restoration of redox and metabolic homeostasis in hepatic tissue. Importantly, the therapeutic efficacy of HST is significantly limited in its free form due to suboptimal solubility and bioavailability; however, nanoparticle-based delivery systems (e.g., Au-mPEG-based formulations) markedly enhance drug solubility, stability, targeted delivery, and tissue accumulation, resulting in improved pharmacokinetic behavior, stronger antioxidant and anti-inflammatory activity, and enhanced anticancer efficacy compared with free HST, thereby supporting their superior translational potential in HCC therapy.

### 6.5 Critical analysis, limitations, and translational perspectives

Current empirical evidence collectively suggests that HST exerts significant anticancer and chemopreventive effects in HCC through the modulation of apoptosis, oxidative stress, inflammatory signaling pathways, mitochondrial dysfunction, lipid metabolism, and the homeostasis of cellular energy. Numerous studies have consistently demonstrated that HST inhibits the proliferation of tumor cells, induces apoptosis mediated by mitochondrial pathways, facilitates cell cycle arrest, and suppresses inflammatory pathways, including NF-κB and TNF-α signaling. Moreover, integrative approaches that combine HST with cisplatin, doxorubicin, sesamin, or nanoparticle-based delivery systems frequently yield synergistic anticancer effects, indicating that HST may augment therapeutic efficacy while concurrently minimizing toxicity in non-cancerous cells. Nevertheless, several critical limitations and inconsistencies persist within the extant literature. The majority of studies predominantly utilized HepG2 cell lines and chemically induced rodent models of HCC, while clinical validation and translational research remain conspicuously absent. A notable degree of heterogeneity was also identified concerning the dosage of HST, duration of treatment, formulation strategies, methods of nanoparticle conjugation, and combinatorial treatment regimens, thereby hindering direct comparisons across various studies. Mechanistic discrepancies were particularly prominent concerning the modulation of ROS and the state of oxidative stress. While numerous investigations have posited that HST induces anticancer effects via ROS-mediated mitochondrial apoptosis and the disruption of redox balance, alternative studies have indicated that its synergistic cytotoxicity may transpire independently of oxidative DNA damage or ROS production, instead involving metabolic reprogramming and the inhibition of glycolysis through the downregulation of HK2 and LDHA. In a similar vein, the therapeutic effects observed appeared to be markedly context-dependent, shaped by the presence of co-administered agents, the cellular metabolic environment, and prevailing redox conditions. Research utilizing xenograft or chemically induced *in vivo* HCC models offers relatively more robust translational evidence compared to isolated *in vitro* cytotoxicity assays; however, the absence of pharmacokinetic assessments, toxicity evaluations, standardized dosing regimens, and clinical trials significantly restricts the current translational relevance of HST. Therefore, additional well-designed preclinical and clinical studies are necessary to clarify the long-term safety, mechanistic specificity, bioavailability, and therapeutic potential of HST-based interventions in HCC. Compared with free HST, nanoparticle-conjugated formulations demonstrated improved bioavailability, stability, and antiproliferative efficacy, highlighting the translational potential of nanoengineered delivery systems in HCC therapy.

### 6.6 Comparative analysis of nanoparticle-based flavonoid delivery systems in HCC

**FIGURE 4:**
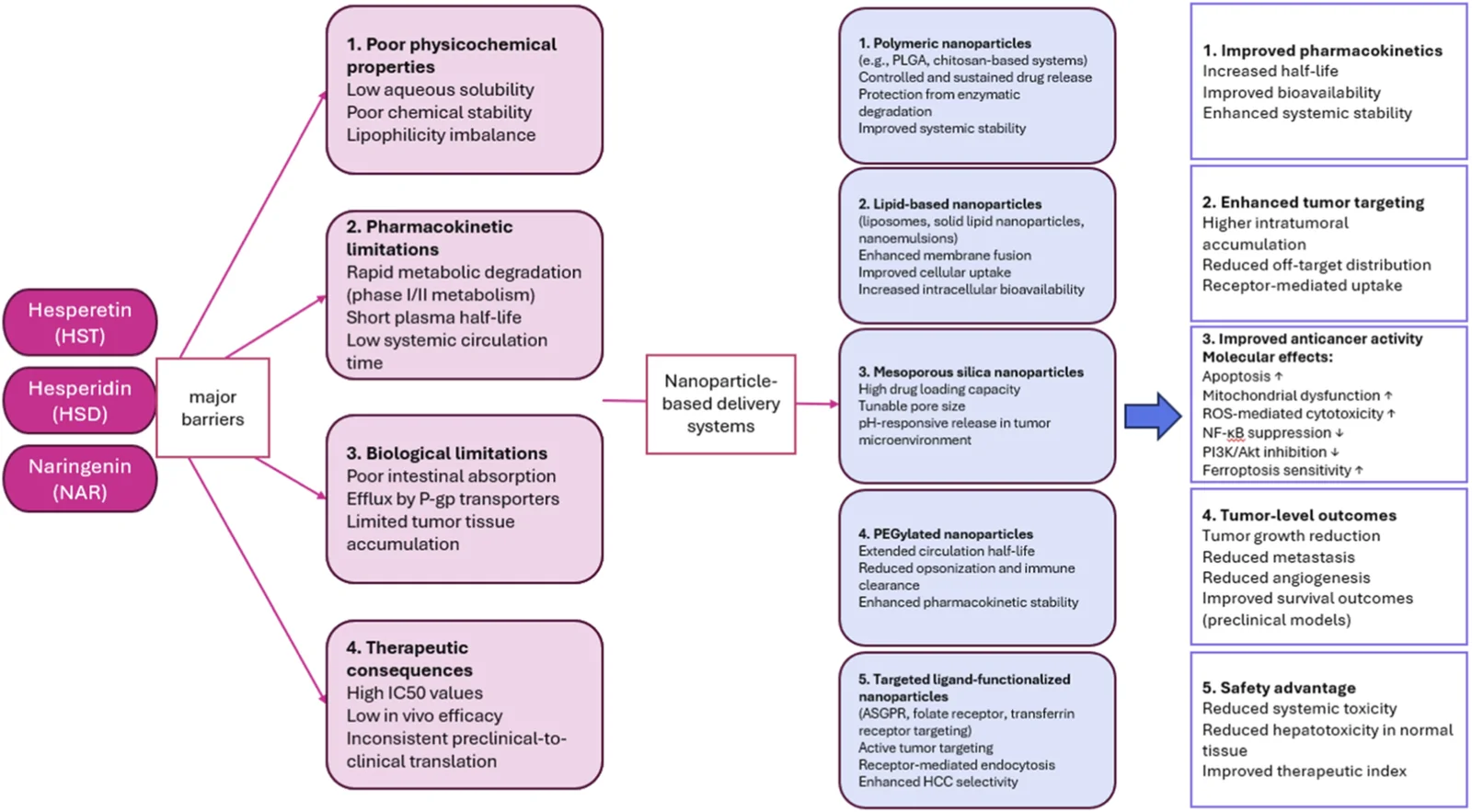
Nanoparticle-based delivery systems of citrus flavonoids in hepatocellular carcinoma (HCC). Schematic representation of nanotechnology-based strategies designed to enhance the therapeutic efficacy of citrus flavonoids, including naringenin (NAR), hesperidin (HSD), and hesperetin (HST), in HCC. The figure illustrates the key limitations of free flavonoids, such as poor aqueous solubility, low bioavailability, rapid metabolic degradation, short circulation time, and limited tumor accumulation, which collectively reduce therapeutic effectiveness. These challenges are addressed through various nanoformulation approaches, including polymeric nanoparticles, lipid-based nanoparticles, mesoporous silica nanoparticles, PEGylated systems, and receptor-targeted nanoparticles (e.g., ASGPR-, folate-, and transferrin-mediated delivery). These nanocarriers improve drug stability, circulation time, cellular uptake, and tumor-specific accumulation while enabling controlled and sustained release. Consequently, nano-encapsulation enhances the anticancer efficacy of flavonoids by promoting apoptosis, inhibiting proliferation, suppressing angiogenesis and metastasis, modulating metabolic reprogramming, and increasing ferroptosis sensitivity in HCC models. Overall, nanoparticle-based delivery systems significantly improve the pharmacokinetic and pharmacodynamic profiles of citrus flavonoids, supporting their translational potential for hepatocellular carcinoma therapy.

The aggregate body of evidence suggests that nanoparticle-mediated delivery systems significantly enhance the therapeutic efficacy of flavonoids in HCC models in comparison to their free counterparts (Figure 4). The primary drawbacks associated with free flavonoids encompass inadequate aqueous solubility, restricted bioavailability, accelerated metabolic degradation, and insufficient intracellular accumulation, all of which may impair therapeutic effectiveness *in vivo*. Strategies involving nanoformulation partially mitigate these shortcomings by augmenting physicochemical stability, extending systemic circulation, facilitating cellular uptake, and enabling sustained or controlled release of the drug. Among the delivery systems examined, polymeric nanoparticles are particularly advantageous due to their sustained release characteristics and their capacity to shield against premature degradation, whereas lipid-based nanoparticles may enhance membrane permeability and intracellular uptake. Conversely, mesoporous silica nanoparticles offer a high drug-loading capacity and allow for tunable surface modifications tailored for controlled delivery applications. Furthermore, pegylated nanoparticle systems enhance pharmacokinetic stability and circulation longevity, thereby improving therapeutic bioavailability and minimizing rapid clearance. Significantly, a number of nanoformulations have exhibited diminished IC50 values, enhanced pro-apoptotic activity, and superior antiproliferative efficacy in comparison to unencapsulated flavonoids. Notwithstanding these encouraging results, a substantial degree of heterogeneity remains evident among the current literature with respect to nanoparticle composition, particle dimensions, surface modifications, dosing methodologies, treatment duration, and experimental models, thus complicating the feasibility of direct comparisons among studies. Moreover, receptor-mediated targeting methodologies continue to be inadequately investigated within the realm of flavonoid-based HCC therapy. Subsequent research endeavors should scrutinize liver-targeting strategies that involve asialoglycoprotein receptor (ASGPR), folate receptor, and transferrin receptor-mediated delivery systems to augment tumor selectivity while concurrently mitigating off-target toxicity. Further pharmacokinetic investigations, prolonged safety evaluations, and clinically pertinent translational models are essential prior to the reliable advancement of flavonoid nanoformulations towards clinical application in the treatment of HCC (Table 4).

**TABLE 4:** Comparative analysis of nanoparticle-based flavonoid delivery systems and their therapeutic advantages in hepatocellular carcinoma.

Flavonoid	Nanoparticle/Delivery system	Main advantages	Proposed mechanistic benefits	Therapeutic outcomes in HCC models
Naringenin (NAR)	Polymeric nanoparticles/nanoencapsulation systems	Improved solubility, stability, intracellular uptake, sustained release	Enhanced intracellular accumulation, improved pharmacokinetics, protection from premature degradation	Lower IC50 values, enhanced apoptosis and autophagy, reduced proliferation and migration
Naringenin (NAR)	Co-encapsulated nanoformulations (e.g., with ibuprofen)	Synergistic delivery, improved bioavailability	Simultaneous modulation of proliferation, apoptosis, angiogenesis, and migration pathways	Enhanced cytotoxicity and antiproliferative activity compared with free compounds
Hesperidin (HSD)	Potential nanoformulation strategies (limited direct evidence)	Improved therapeutic persistence and intracellular delivery	Enhanced physicochemical stability and prolonged release behavior	Potential enhancement of antiproliferative and pro-apoptotic effects
Hesperetin (HST)	Pegylated gold nanoparticles (Au-mPEG (5000)-S-HST NPs)	Improved bioavailability, circulation time, stability, and release profile	Enhanced systemic persistence, improved intracellular delivery, reduced premature degradation	Stronger suppression of hepatocarcinogenesis, oxidative stress, inflammatory signaling, and proliferation compared with free HST
Flavonoid combinations	Nanoparticle-assisted combinational delivery systems	Enhanced synergistic efficacy and reduced systemic toxicity	Improved co-delivery efficiency and tumor-cell exposure	Greater apoptotic induction and tumor-growth inhibition
General flavonoid nanoformulations	Mesoporous silica nanoparticles	High loading capacity and tunable surface modification	Controlled release and potential targeted delivery	Improved therapeutic delivery efficiency and translational potential
General flavonoid nanoformulations	Lipid-based nanoparticles	Improved membrane permeability and cellular uptake	Enhanced intracellular internalization and bioavailability	Increased anticancer activity and therapeutic persistence
General flavonoid nanoformulations	Polymeric nanoparticles	Sustained release and protection from degradation	Improved pharmacokinetic stability and prolonged drug exposure	Enhanced antiproliferative efficacy and reduced drug loss

### 6.7 Synergistic therapeutic potential of combined flavonoid strategies in HCC

The integrated therapeutic utilization of NAR, HSD, and HST may signify a promising multitargeted approach for the management of HCC, as these flavonoids modulate complementary molecular pathways that are implicated in tumor progression. Collectively, the existing body of evidence indicates that these compounds regulate apoptosis, ferroptosis, oxidative stress, inflammatory signaling, angiogenesis, metabolic reprogramming, and metastatic behavior via pathways that encompass PI3K/Akt, NF-κB, Wnt/β-catenin, MAPK, AMPK/PGC1α, and JAK/STAT signaling. Notably, their partially overlapping yet mechanistically distinct activities may confer synergistic anticancer effects when administered in combination. NAR appears particularly efficacious in modulating ferroptosis, angiogenesis, immune evasion, and metabolic signaling, whereas HSD exhibits robust anti-inflammatory, anti-invasive, and epigenetic regulatory properties. Conversely, HST prominently targets mitochondrial apoptosis, oxidative stress homeostasis, and inflammatory cascades. Consequently, combinatorial flavonoid strategies may concurrently address multiple hallmarks of HCC progression, potentially augmenting tumor suppression while diminishing the probability of therapeutic resistance. Moreover, the integration of flavonoids with chemotherapeutic agents or nanoparticle-mediated delivery systems has the potential to augment intracellular drug accumulation, amplify therapeutic sensitivity, and mitigate systemic toxicity. However, empirical evidence directly assessing the efficacy of triple-flavonoid combinational therapy in HCC is still scant, necessitating further research to elucidate optimal dosing regimens, pharmacokinetic interactions, long-term safety profiles, and translational relevance.

### 6.8 Nanotechnology-based targeted delivery strategies for reducing toxicity in HCC

Nanotechnology-based delivery systems may also reduce the systemic toxicity and adverse effects commonly associated with conventional HCC therapies by enabling more selective drug accumulation within tumor tissues while limiting exposure to normal cells. Nanoformulations can improve the therapeutic index of flavonoids through controlled release behavior, enhanced intracellular uptake, and reduced premature drug degradation. In particular, receptor-mediated targeting strategies involving ASGPR, folate receptor, and transferrin receptor pathways may enhance selective delivery to HCC cells, thereby minimizing off-target toxicity and improving therapeutic precision. Furthermore, pegylated and ligand-functionalized nanoparticles may prolong systemic circulation and improve tumor retention, contributing to greater therapeutic efficacy with potentially lower systemic side effects.

## 7 Challenges and limitations

### 7.1 Poor bioavailability

Despite the encouraging anticancer properties associated with citrus flavonoids, their clinical application is considerably constrained due to inadequate bioavailability and suboptimal pharmacokinetic profiles. Numerous investigations have underscored that native NAR demonstrates poor solubility and restricted accumulation at tumor loci, subsequently diminishing its therapeutic effectiveness. This constraint has been explicitly addressed through research utilizing NARNPs, wherein nanoformulation substantially augmented cytotoxicity in comparison to unmodified NAR. In a similar vein, zein/sodium caseinate nanoparticles co-encapsulated with NAR exhibited enhanced antiproliferative and anti-angiogenic properties, thereby indicating that delivery systems are pivotal in surmounting pharmacokinetic obstacles. Analogous challenges have been noted for HST and HSD, wherein nanoparticle-based delivery systems markedly improved solubility, bioavailability, and anticancer effectiveness *in vivo*. Overall, the prevailing body of evidence robustly indicates that nano-delivery systems, encapsulation methodologies, and combinatorial formulations are imperative for addressing bioavailability limitations; however, there remains a conspicuous absence of standardized delivery platforms.

### 7.2 Dose optimization

Another significant constraint is the inadequacy of precisely delineated therapeutic dosing protocols for these flavonoids. The studies reviewed exhibit considerable variability in effective concentrations across different experimental models, thereby complicating the translation of findings into clinical applications. For instance, NAR demonstrated dose-dependent dual effects, wherein elevated concentrations prompted apoptosis and inhibited cellular proliferation, while reduced concentrations appeared to diminish the efficacy of chemotherapeutic agents. HSD and HST displayed extensive IC50 ranges, thereby raising concerns regarding the attainment of therapeutically relevant plasma concentrations in human subjects. Consequently, there is an urgent necessity for systematic dose–response investigations, pharmacodynamic profiling, and the establishment of standardized dosing frameworks to ensure safety and optimize therapeutic efficacy.

### 7.3 Translational gaps

A significant obstacle in the progression of citrus-derived flavonoids, including NAR, HSD, and HST, towards clinical utilization in HCC lies in the considerable disparity between preclinical findings and their applicability to human subjects. The majority of the extant literature is derived from *in vitro* investigations utilizing liver cancer cell lines and *in vivo* animal studies, particularly those involving chemically induced HCC models such as DEN and TAA. Although these investigations consistently reveal pronounced anticancer properties, they fail to adequately represent the complexity and heterogeneity characteristic of human HCC. One significant concern pertains to tumor heterogeneity, as human HCC displays a wide array of genetic, epigenetic, and metabolic characteristics that are insufficiently represented in experimental models. Furthermore, interspecies variations in metabolic processes, pharmacokinetics, and immune responses may profoundly affect the efficacy and safety profiles of these compounds, thus constraining the predictive validity of animal research. Importantly, although multiple molecular signaling pathways discussed throughout this review—including PI3K/Akt, Wnt/β-catenin, MAPK, NF-κB, AMPK, and JAK2/STAT3—are well-established drivers of hepatocarcinogenesis and clinically relevant therapeutic targets in HCC, current evidence regarding citrus flavonoids remains predominantly preclinical. Consequently, the translational significance of these mechanistic findings requires further validation in human subjects. Although numerous investigations have elucidated the synergistic effects of these flavonoids when administered in conjunction with standard therapies such as cisplatin, doxorubicin, sorafenib, or immune checkpoint inhibitors, the clinical viability of these combinations remains ambiguous due to the absence of rigorously controlled human clinical trials. Moreover, there exists a notable deficiency of information concerning the long-term safety, optimal routes of administration, and potential drug–drug interactions within clinical environments. Additional challenges include poor oral bioavailability, limited systemic stability, variable intestinal metabolism, and insufficient data regarding clinically achievable therapeutic concentrations in humans. The lack of standardized methodologies for assessing pharmacodynamics and therapeutic outcomes further exacerbates the discrepancies observed across various studies. Notably, although nanoformulations and sophisticated drug delivery systems have demonstrated potential in enhancing bioavailability and targeting efficacy in preclinical settings, their scalability, regulatory sanction, and clinical feasibility remain to be thoroughly validated. In summary, these constraints highlight the pressing necessity for meticulously designed clinical trials, exhaustive pharmacokinetic and toxicological assessments, and more physiologically relevant models to effectively bridge the divide between experimental results and clinical application. Addressing these obstacles will be crucial for substantiating the therapeutic efficacy of citrus flavonoids and facilitating their incorporation into evidence-based treatment paradigms for HCC.

## 8 Future perspectives and research directions

Future investigations concerning citrus flavonoids, including NAR, HSD, and HST, in the context of HCC ought to concentrate on the formulation of multi-target therapeutic modalities that encapsulate the intricate and heterogeneous characteristics of tumor biology. An increasing body of literature suggests that these phytochemicals concurrently influence various oncogenic signaling pathways, while also modulating critical biological processes, including apoptosis, ferroptosis, angiogenesis, and tumor immunity. This multifaceted activity establishes citrus flavonoids as viable candidates for network-based therapeutic approaches instead of singular target interventions. Subsequent research endeavors should aspire to amalgamate systems biology methodologies, omics technologies, and pathway interaction assessments to enhance comprehension of the interplay among these signaling networks and to elucidate pivotal nodes for therapeutic targeting. Furthermore, strategies involving structural modification and nanoformulation may augment their capacity to concurrently engage multiple molecular pathways with enhanced therapeutic efficacy. Another auspicious avenue for exploration entails the integration of personalized medicine methodologies. In light of the considerable heterogeneity observed in HCC at the genetic, epigenetic, and metabolic dimensions, stratifying patients according to their molecular signatures could markedly enhance therapeutic efficacy. Biomarkers associated with metabolic reprogramming, oxidative stress, and activation of specific pathways may facilitate the identification of patient subpopulations that are predisposed to respond favorably to interventions based on citrus flavonoids. Additionally, the amalgamation of pharmacogenomics and metabolomics has the potential to optimize dosing regimens and reduce variability in treatment responses. Consequently, forthcoming clinical investigations should prioritize frameworks of precision oncology, wherein citrus flavonoids are customized to align with the unique profiles of individual patients. The integration of citrus flavonoids with established therapeutic modalities signifies a notably auspicious pathway for enhancing treatment effectiveness and surmounting pharmacological resistance. Preliminary investigations provide evidence that these bioactive compounds can augment the susceptibility of HCC cells to chemotherapeutic agents including cisplatin, doxorubicin, and sorafenib, as well as to ferroptosis inducers, through the modulation of oxidative stress, metabolic pathways, and survival signaling mechanisms. Moreover, their capacity to downregulate immune checkpoints and modify the tumor microenvironment indicates a prospective synergy with immunotherapeutic strategies. In summary, the progression of citrus flavonoids towards clinical implementation in the context of HCC will necessitate a multidisciplinary strategy that amalgamates multi-target pharmacology, precision medicine, and judicious combination therapies. Ongoing endeavors in translational research, bolstered by meticulously designed clinical trials, will be imperative to fully actualize their therapeutic promise.

## 9 Conclusion

Citrus-derived flavonoids, including NAR, HSD, and HST, exhibit notable anticancer activities in HCC based on preclinical *in vitro* and *in vivo* evidence. These compounds have been shown to modulate key biological processes involved in tumor progression, including cell proliferation, apoptosis, oxidative stress, and metastasis, primarily through regulation of major oncogenic signaling pathways such as PI3K/Akt, Wnt/β-catenin, MAPK, and JAK2/STAT3. In addition, experimental studies suggest that these flavonoids may enhance the activity of conventional chemotherapeutic agents and demonstrate improved pharmacological properties when delivered via advanced formulation strategies in preclinical models. However, the current evidence is largely limited to experimental systems, and there is a lack of robust clinical data to support their therapeutic efficacy in humans. Therefore, while citrus flavonoids represent promising bioactive compounds in experimental HCC research, their clinical relevance remains to be established. Further well-designed clinical, pharmacokinetic, and toxicological studies are necessary to validate their safety, efficacy, and translational potential before any clinical application can be considered.

## References

[B1] Ahmed O. M. Ahmed A. A. Fahim H. I. Zaky M. Y. (2022). Quercetin and naringenin abate diethylnitrosamine/acetylaminofluorene-induced hepatocarcinogenesis in wistar rats: the roles of oxidative stress, inflammation and cell apoptosis. Drug Chem. Toxicol. 45, 262–273. 10.1080/01480545.2019.1683187 31665932

[B2] Almoallim H. S. Aljawdah H. M. Bharathi M. Manickam R. Abudoleh S. M. Hussein-Al-Ali S. H. (2024). Fabrication of ibuprofen/naringenin-coloaded into zein/sodium caseinate nanoparticles: evaluation of antiproliferative activity and apoptosis induction in liver cancer cells. J. Biomater. Sci. Polym. Ed. 35, 2703–2722. 10.1080/09205063.2024.2391653 39217620

[B3] Anstee Q. M. Reeves H. L. Kotsiliti E. Govaere O. Heikenwalder M. (2019). From NASH to HCC: current concepts and future challenges. Nat. Rev. Gastroenterol. Hepatol. 16, 411–428. 10.1038/s41575-019-0145-7 31028350

[B4] Artanti A. N. Jenie R. I. Rumiyati R. Meiyanto E. (2024). Hesperidin and diosmin increased cytotoxic activity cisplatin on hepatocellular carcinoma and protect kidney cells senescence. Asian Pac J. Cancer Prev. 25, 4247–4255. 10.31557/apjcp.2024.25.12.4247 39733416 PMC12008348

[B5] Artanti A. N. Jenie R. I. Rumiyati R. Hermawan A. Jun-Ya K. Hanifa M. (2026). Investigation of the anticancer effects and senescence induction of hesperetin combined with cisplatin in hepatocellular carcinoma and embryonic fibroblast cell lines. Asian Pac J. Cancer Prev. 27, 557–568. 10.31557/apjcp.2026.27.2.557 41660913 PMC13495531

[B6] Arul D. Subramanian P. (2013). Inhibitory effect of naringenin (citrus flavonone) on N-nitrosodiethylamine induced hepatocarcinogenesis in rats. Biochem. Biophys. Res. Commun. 434, 203–209. 10.1016/j.bbrc.2013.03.039 23523793

[B7] Banjerdpongchai R. Wudtiwai B. Khaw-On P. Rachakhom W. Duangnil N. Kongtawelert P. (2016). Hesperidin from citrus seed induces human hepatocellular carcinoma HepG2 cell apoptosis via both mitochondrial and death receptor pathways. Tumour Biol. 37, 227–237. 10.1007/s13277-015-3774-7 26194866 PMC4841854

[B8] Bhardwaj R. Sharma N. Rajput S. K. (2026). “Liver cancer: epidemiology, prevalence and advanced modeling techniques,” in Modeling Prevalent Cancers. 1st ed. Boca Raton (FL): CRC Press, 221–254.

[B9] Chen Y. Bai M. Liu M. Zhang Z. Jiang C. Li K. (2025). Metabolic reprogramming in lung cancer: Hallmarks, mechanisms, and targeted strategies to overcome immune resistance. Cancer Med. 14, e71317. 10.1002/cam4.71317 41163383 PMC12571976

[B10] Cheng H. Wang M. Su J. Li Y. Long J. Chu J. (2022). Lipid metabolism and cancer. Life 12, 784. 10.3390/life12060784 35743814 PMC9224822

[B11] D'Archivio M. Filesi C. Varì R. Scazzocchio B. Masella R. (2010). Bioavailability of the polyphenols: status and controversies. Int. J. Mol. Sci. 11, 1321–1342. 10.3390/ijms11041321 20480022 PMC2871118

[B12] Dixon S. J. Lemberg K. M. Lamprecht M. R. Skouta R. Zaitsev E. M. Gleason C. E. (2012). Ferroptosis: an iron-dependent form of nonapoptotic cell death. Cell 149, 1060–1072. 10.1016/j.cell.2012.03.042 22632970 PMC3367386

[B13] Dodson M. Castro-Portuguez R. Zhang D. D. (2019). NRF2 plays a critical role in mitigating lipid peroxidation and ferroptosis. Redox Biol. 23, 101107. 10.1016/j.redox.2019.101107 30692038 PMC6859567

[B14] Elnawasany S. Haggag Y. A. Shalaby S. M. Soliman N. A. El Saadany A. A. Ibrahim M. A. A. (2023). Anti-cancer effect of nano-encapsulated boswellic acids, curcumin and naringenin against HepG-2 cell line. BMC Complement. Med. Ther. 23, 270. 10.1186/s12906-023-04096-4 37516826 PMC10386659

[B15] Elwan A. G. Mohamed T. M. Beltagy D. M. El Gamal D. M. (2025). The therapeutic role of naringenin nanoparticles on hepatocellular carcinoma. BMC Pharmacol. Toxicol. 26, 3. 10.1186/s40360-024-00823-w 39754228 PMC11697747

[B16] Farhan M. Rizvi A. Aatif M. Ahmad A. (2023). Current understanding of flavonoids in cancer therapy and prevention. Metabolites 13, 481. 10.3390/metabo13040481 37110140 PMC10142845

[B17] Fernández-Bedmar Z. Anter J. Alonso-Moraga A. Martín de Las Mulas J. Millán-Ruiz Y. Guil-Luna S. (2017). Demethylating and anti-hepatocarcinogenic potential of hesperidin, a natural polyphenol of citrus juices. Mol. Carcinog. 56, 1653–1662. 10.1002/mc.22621 28130850

[B18] Flavin R. Peluso S. Nguyen P. L. Loda M. (2010). Fatty acid synthase as a potential therapeutic target in cancer. Future Oncol. 6, 551–562. 10.2217/fon.10.11 20373869 PMC3197858

[B19] Foglia B. Beltrà M. Sutti S. Cannito S. (2023). Metabolic reprogramming of HCC: a new microenvironment for immune responses. Int. J. Mol. Sci. 24, 7463. 10.3390/ijms24087463 37108625 PMC10138633

[B20] Forner A. Reig M. Bruix J. (2018). Hepatocellulars carcinoma. Lancet 391, 1301–1314. 10.1016/S0140-6736(18)30010-2 29307467

[B21] Gokuladhas K. Jayakumar S. Rajan B. Elamaran R. Pramila C. S. Gopikrishnan M. (2016). Exploring the potential role of chemopreventive agent, hesperetin conjugated pegylated gold nanoparticles in diethylnitrosamine-induced hepatocellular carcinoma in Male wistar albino rats. Indian J. Clin. Biochem. 31, 171–184. 10.1007/s12291-015-0520-2 27069325 PMC4820423

[B22] Hanahan D. (2022). Hallmarks of cancer: new dimensions. Cancer Discov. 12, 31–46. 10.1158/2159-8290.cd-21-1059 35022204

[B23] Huang D. Q. Mathurin P. Cortez-Pinto H. Loomba R. (2023). Global epidemiology of alcohol-associated cirrhosis and HCC: trends, projections and risk factors. Nat. Rev. Gastroenterol. Hepatol. 20, 37–49. 10.1038/s41575-022-00688-6 36258033 PMC9579565

[B24] Jones S. F. Infante J. R. (2015). Molecular pathways: fatty acid synthase. Clin. Cancer Res. 21, 5434–5438. 10.1158/1078-0432.CCR-15-0126 26519059

[B25] Kang Q. Gong J. Wang M. Wang Q. Chen F. Cheng K. W. (2019). 6-C-(E-Phenylethenyl)Naringenin attenuates the stemness of hepatocellular carcinoma cells by suppressing Wnt/β-Catenin signaling. J. Agric. Food Chem. 67, 13939–13947. 10.1021/acs.jafc.9b05733 31769973

[B26] Khuanphram N. Taya S. Kongtawelert P. Wongpoomchai R. (2021). Sesame extract promotes chemopreventive effect of hesperidin on early phase of diethylnitrosamine-initiated hepatocarcinogenesis in rats. Pharmaceutics 13, 1687. 10.3390/pharmaceutics13101687 34683980 PMC8538859

[B27] Kim J. Y. Han E. H. Shin D. W. Jeong T. C. Lee E. S. Woo E. R. (2004). Suppression of CYP1A1 expression by naringenin in murine Hepa-1c1c7 cells. Arch. Pharm. Res. 27, 857–862. 10.1007/BF02980179 15460448

[B28] Korga A. Ostrowska M. Jozefczyk A. Iwan M. Wojcik R. Zgorka G. (2019). Apigenin and hesperidin augment the toxic effect of doxorubicin against HepG2 cells. BMC Pharmacol. Toxicol. 20, 22. 10.1186/s40360-019-0301-2 31053173 PMC6499973

[B29] Krishnan G. Subramaniyan J. Chengalvarayan Subramani P. Muralidharan B. Thiruvengadam D. (2017). Hesperetin conjugated PEGylated gold nanoparticles exploring the potential role in anti-inflammation and anti-proliferation during diethylnitrosamine-induced hepatocarcinogenesis in rats. Asian J. Pharm. Sci. 12, 442–455. 10.1016/j.ajps.2017.04.001 32104357 PMC7032104

[B30] Lee K. H. Yeh M. H. Kao S. T. Hung C. M. Liu C. J. Huang Y. Y. (2010). The inhibitory effect of hesperidin on tumor cell invasiveness occurs via suppression of activator protein 1 and nuclear factor-kappaB in human hepatocellular carcinoma cells. Toxicol. Lett. 194, 42–49. 10.1016/j.toxlet.2010.01.021 20138977

[B31] Li Y. Z. Deng J. Zhang X. D. Li D. Y. Su L. X. Li S. (2024). Naringenin enhances the efficacy of ferroptosis inducers by attenuating aerobic glycolysis by activating the AMPK-PGC1α signalling axis in liver cancer. Heliyon 10, e32288. 10.1016/j.heliyon.2024.e32288 38912485 PMC11190665

[B32] Lin X. Kong L. N. Huang C. Ma T. T. Meng X. M. He Y. (2015). Hesperetin derivative-7 inhibits PDGF-BB-induced hepatic stellate cell activation and proliferation by targeting Wnt/β-catenin pathway. Int. Immunopharmacol. 25, 311–320. 10.1016/j.intimp.2015.02.009 25701506

[B33] Liskova A. Samec M. Koklesova L. Brockmueller A. Zhai K. Abdellatif B. (2021). Flavonoids as an effective sensitizer for anti-cancer therapy: insights into multi-faceted mechanisms and applicability towards individualized patient profiles. EPMA J. 12, 155–176. 10.1007/s13167-021-00242-5 34025826 PMC8126506

[B34] Liu S. Zhang X. Wang W. Li X. Sun X. Zhao Y. (2024). Metabolic reprogramming and therapeutic resistance in primary and metastatic breast cancer. Mol. Cancer 23, 261. 10.1186/s12943-024-02165-x 39574178 PMC11580516

[B35] Llovet J. M. Beaugrand M. (2003). Hepatocellular carcinoma: present status and future prospects. J. Hepatol. 38 (Suppl. 1), S136–S149. 10.1016/s0168-8278(02)00432-4 12591191

[B36] Llovet J. M. Kelley R. K. Villanueva A. Singal A. G. Pikarsky E. Roayaie S. (2021). Hepatocellular carcinoma. Nat. Rev. Dis. Prim. 7, 6. 10.1038/s41572-020-00240-3 33479224 PMC13537433

[B37] Mahmoud A. M. Mohammed H. M. Khadrawy S. M. Galaly S. R. (2017). Hesperidin protects against chemically induced hepatocarcinogenesis via modulation of Nrf2/ARE/HO-1, PPARγ and TGF-β1/Smad3 signaling, and amelioration of oxidative stress and inflammation. Chem. Biol. Interact. 277, 146–158. 10.1016/j.cbi.2017.09.015 28935427

[B38] Manach C. Scalbert A. Morand C. Rémésy C. Jiménez L. (2004). Polyphenols: food sources and bioavailability. Am. J. Clin. Nutr. 79, 727–747. 10.1093/ajcn/79.5.727 15113710

[B39] Mauro E. Ferrer-Fàbrega J. Sauri T. Soler A. Cobo A. Burrel M. (2023). New challenges in the management of cholangiocarcinoma: the role of liver transplantation, locoregional therapies, and systemic therapy. Cancers 15, 1244. 10.3390/cancers15041244 36831586 PMC9953927

[B40] McGlynn K. A. Petrick J. L. El-Serag H. B. (2021). Epidemiology of hepatocellular carcinoma. Hepatology 73 (Suppl. 1), 4–13. 10.1002/hep.31288 32319693 PMC7577946

[B41] Mo'men Y. S. Hussein R. M. Kandeil M. A. (2019). Involvement of PI3K/Akt pathway in the protective effect of hesperidin against a chemically induced liver cancer in rats. J. Biochem. Mol. Toxicol. 33, e22305. 10.1002/jbt.22305 30779474

[B42] Montané X. Kowalczyk O. Reig-Vano B. Bajek A. Roszkowski K. Tomczyk R. (2020). Current perspectives of the applications of polyphenols and flavonoids in cancer therapy. Molecules 25. 10.3390/molecules25153342 32717865 PMC7435624

[B43] Naeem A. Hu P. Yang M. Zhang J. Liu Y. Zhu W. (2022). Natural products as anticancer agents: current status and future perspectives. Molecules 27, 8367. 10.3390/molecules27238367 36500466 PMC9737905

[B44] Navarro C. Ortega Á. Santeliz R. Garrido B. Chacín M. Galban N. (2022). Metabolic reprogramming in cancer cells: emerging molecular mechanisms and novel therapeutic approaches. Pharmaceutics 14, 1303. 10.3390/pharmaceutics14061303 35745875 PMC9227908

[B45] Naz H. Tarique M. Ahamad S. Alajmi M. F. Hussain A. Rehman M. T. (2019). Hesperidin-CAMKIV interaction and its impact on cell proliferation and apoptosis in the human hepatic carcinoma and neuroblastoma cells. J. Cell Biochem. 120, 15119–15130. 10.1002/jcb.28774 31021496

[B46] Panche A. N. Diwan A. D. Chandra S. R. (2016). Flavonoids: an overview. J. Nutr. Sci. 5, e47. 10.1017/jns.2016.41 28620474 PMC5465813

[B47] Pandey P. Khan F. (2021). A mechanistic review of the anticancer potential of hesperidin, a natural flavonoid from citrus fruits. Nutr. Res. 92, 21–31. 10.1016/j.nutres.2021.05.011 34273640

[B48] Park S. Hall M. N. (2025). Metabolic reprogramming in hepatocellular carcinoma: mechanisms and therapeutic implications. Exp. Mol. Med. 57, 515–523. 10.1038/s12276-025-01415-2 40025169 PMC11958682

[B49] Pavlova N. N. Thompson C. B. (2016). The emerging hallmarks of cancer metabolism. Cell Metab. 23, 27–47. 10.1016/j.cmet.2015.12.006 26771115 PMC4715268

[B50] Phucharoenrak P. Muangnoi C. Trachootham D. (2023). Metabolomic analysis of phytochemical compounds from ethanolic extract of lime (citrus aurantifolia) peel and its anti-cancer effects against human hepatocellular carcinoma cells. Molecules 28, 2965. 10.3390/molecules28072965 37049726 PMC10095956

[B51] Rani N. Bharti S. Krishnamurthy B. Bhatia J. Sharma C. Kamal M. A. (2016). Pharmacological properties and therapeutic potential of naringenin: a citrus flavonoid of pharmaceutical promise. Curr. Pharm. Des. 22, 4341–4359. 10.2174/1381612822666160530150936 27238365

[B52] Reczek C. R. Chandel N. S. (2015). ROS-Dependent signal transduction. Curr. Opin. Cell Biol. 33, 8–13. 10.1016/j.ceb.2014.09.010 25305438 PMC4380867

[B53] Ringelhan M. Pfister D. O'Connor T. Pikarsky E. Heikenwalder M. (2018). The immunology of hepatocellular carcinoma. Nat. Immunol. 19, 222–232. 10.1038/s41590-018-0044-z 29379119

[B54] Samodien S. de Kock M. Joubert E. de Beer D. Kriel J. Gelderblom W. C. A. (2024). Autophagy-induced cell death by aqueous and polyphenol-enriched extracts of honeybush (cyclopia spp.) in liver and Colon cancer cells. Food Sci. Nutr. 12, 5647–5662. 10.1002/fsn3.4214 39139978 PMC11317699

[B55] Sapisochin G. Bruix J. (2017). Liver transplantation for hepatocellular carcinoma: outcomes and novel surgical approaches. Nat. Rev. Gastroenterol. Hepatol. 14, 203–217. 10.1038/nrgastro.2016.193 28053342

[B56] Schirrmacher V. (2020). Mitochondria at work: new insights into regulation and dysregulation of cellular energy supply and metabolism. Biomedicines 8. 10.3390/biomedicines8110526 33266387 PMC7700424

[B57] Sharma S. Mishra A. Ramniwas S. Pandey P. (2025). An updated review summarizing the anticancer potential of naringenin. Endocr. Metab. Immune Disord. Drug Targets 25, 364–376. 10.2174/0118715303308238240705061522 39005120

[B58] Sharma A. Kumar A. Chaudhary R. P. Singh A. K. Singh H. Kumar P. (2026). Unrevealing the multifaceted role of resveratrol as potential antioxidant and anticancer agent: molecular mechanism and future perspectives. Chem. Biodivers. 23, e03280. 10.1002/cbdv.202403280 41790488

[B59] Shine V. Anuja G. Pradeep S. Suja S. (2018). Molecular interaction of naringin and its metabolite naringenin to human liver fibrosis proteins: an *in silico* approach. Pharmacogn. Mag. 14, 102. 10.4103/pm.pm_453_17

[B60] Sung H. Ferlay J. Siegel R. L. Laversanne M. Soerjomataram I. Jemal A. (2021). Global cancer statistics 2020: GLOBOCAN estimates of incidence and mortality worldwide for 36 cancers in 185 countries. CA Cancer J. Clin. 71, 209–249. 10.3322/caac.21660 33538338

[B61] Tang D. Chen X. Kang R. Kroemer G. (2021). Ferroptosis: molecular mechanisms and health implications. Cell Res. 31, 107–125. 10.1038/s41422-020-00441-1 33268902 PMC8026611

[B62] Vander Heiden M. G. Cantley L. C. Thompson C. B. (2009). Understanding the warburg effect: the metabolic requirements of cell proliferation. Science 324, 1029–1033. 10.1126/science.1160809 19460998 PMC2849637

[B63] Wang M. Qu L. Du X. Song P. Ng J. P. L. Wong V. K. W. (2024a). Natural products and derivatives targeting metabolic reprogramming in colorectal cancer: a comprehensive review. Metabolites 14, 490. 10.3390/metabo14090490 39330497 PMC11433951

[B64] Wang H. Liang J. Wang Y. Zheng J. Liu Y. Zhao Y. (2024b). Exploring the effects of naringin on oxidative stress-impaired osteogenic differentiation via the Wnt/β-catenin and PI3K/Akt pathways. Sci. Rep. 14, 14047. 10.1038/s41598-024-64952-2 38890371 PMC11189479

[B65] Wu Y. Lin Y. Xu S. Su D. Yang H. Tang L. (2025a). Mechanisms of hesperetin in treating metabolic dysfunction-associated steatosis liver disease via network pharmacology and *in vitro* experiments. Open Med. 20, 20251215. 10.1515/med-2025-1215 40520339 PMC12163577

[B66] Wu W. Qiu X. Ye X. Zhang Z. Xu S. Yao X. (2025b). Naringenin: a potential therapeutic agent for modulating angiogenesis and immune response in hepatocellular carcinoma. J. Pharm. Anal. 15, 101254. 10.1016/j.jpha.2025.101254 41079786 PMC12513016

[B67] Xu Z. Jia Y. Liu J. Ren X. Yang X. Xia X. (2022). Naringenin and quercetin exert contradictory cytoprotective and cytotoxic effects on tamoxifen-induced apoptosis in HepG2 cells. Nutrients 14, 5394. 10.3390/nu14245394 36558554 PMC9783584

[B68] Yeh M. H. Kao S. T. Hung C. M. Liu C. J. Lee K. H. Yeh C. C. (2009). Hesperidin inhibited acetaldehyde-induced matrix metalloproteinase-9 gene expression in human hepatocellular carcinoma cells. Toxicol. Lett. 184, 204–210. 10.1016/j.toxlet.2008.11.018 19110045

[B69] Yen H. R. Liu C. J. Yeh C. C. (2015). Naringenin suppresses TPA-Induced tumor invasion by suppressing multiple signal transduction pathways in human hepatocellular carcinoma cells. Chem. Biol. Interact. 235, 1–9. 10.1016/j.cbi.2015.04.003 25866363

[B70] Yumnam S. Hong G. E. Raha S. Saralamma V. V. Lee H. J. Lee W. S. (2016). Mitochondrial dysfunction and Ca(2+) overload contributes to hesperidin induced paraptosis in hepatoblastoma cells, HepG2. J. Cell Physiol. 231, 1261–1268. 10.1002/jcp.25222 26492105

[B71] Zaghloul R. A. Elsherbiny N. M. Kenawy H. I. El-Karef A. Eissa L. A. El-Shishtawy M. M. (2017). Hepatoprotective effect of hesperidin in hepatocellular carcinoma: involvement of wnt signaling pathways. Life Sci. 185, 114–125. 10.1016/j.lfs.2017.07.026 28754618

[B72] Zhang J. Song J. Wu D. Wang J. Dong W. (2015). Hesperetin induces the apoptosis of hepatocellular carcinoma cells via mitochondrial pathway mediated by the increased intracellular reactive oxygen species, ATP and calcium. Med. Oncol. 32, 101. 10.1007/s12032-015-0516-z 25737432

[B73] Zhang M. Lai J. Wu Q. Lai J. Su J. Zhu B. (2023). Naringenin induces HepG2 cell apoptosis via ROS-mediated JAK-2/STAT-3 signaling pathways. Molecules 28, 4506. 10.3390/molecules28114506 37298981 PMC10254813

[B74] Zhang Z. Wang H. Liu X. He M. Lin Y. (2025). Exploring the mechanism of naringenin in the treatment of hepatocellular carcinoma based on mRNA sequencing and experimental validation. Sci. Rep. 15, 23109. 10.1038/s41598-025-09013-y 40593127 PMC12218334

